# A genome-wide analysis of coatomer protein (COP) subunits of apicomplexan parasites and their evolutionary relationships

**DOI:** 10.1186/s12864-019-5463-1

**Published:** 2019-01-31

**Authors:** K. M. Kaderi Kibria, Jannatul Ferdous, Rahila Sardar, Ashutosh Panda, Dinesh Gupta, Asif Mohmmed, Pawan Malhotra

**Affiliations:** 1 Department of Biotechnology and Genetic Engineering, Mawlana Bhashani Science and Technology University, Santosh, Tangail, 1902 Bangladesh; 2 Translational Bioinformatics Group, International Centre for Genetic Engineering and Biotechnology, P. O. Box 10504, Aruna Asaf Ali Marg, New Delhi, 110067 India; 3 Department of Microbiology, All India Institute of Medical Science, New Delhi, 29 India; 4 Malaria Group, International Centre for Genetic Engineering and Biotechnology, P. O. Box 10504, Aruna Asaf Ali Marg, New Delhi, 110067 India

**Keywords:** COPI, COPII, Apicomplexa, Golgi, Endoplasmic reticulum, Protein trafficking

## Abstract

**Background:**

Protein secretion is an essential process in all eukaryotes including organisms belonging to the phylum Apicomplexa, which includes many intracellular parasites. The apicomplexan parasites possess a specialized collection of secretory organelles that release a number of proteins to facilitate the invasion of host cells and some of these proteins also participate in immune evasion. Like in other eukaryotes, these parasites possess a series of membrane-bound compartments, namely the endoplasmic reticulum (ER), the intermediate compartments (IC) or vesicular tubular clusters (VTS) and Golgi complex through which proteins pass in a sequential and vectorial fashion. Two sets of proteins; COPI and COPII are important for directing the sequential transfer of material between the ER and Golgi complex.

**Results:**

Here, using in silico approaches, we identify the components of COPI and COPII complexes in the genome of apicomplexan organisms. The results showed that the COPI and COPII protein complexes are conserved in most apicomplexan genomes with few exceptions. Diversity among the components of COPI and COPII complexes in apicomplexan is either due to the absence of a subunit or due to the difference in the number of protein domains. For example, the COPI epsilon subunit and COPII sec13 subunit is absent in *Babesia bovis, Theileria parva,* and *Theileria annulata* genomes. Phylogenetic and domain analyses for all the proteins of COPI and COPII complexes was performed to predict their evolutionary relationship and functional significance.

**Conclusions:**

The study thus provides insights into the apicomplexan COPI and COPII coating machinery, which is crucial for parasites secretory network needed for the invasion of host cells.

**Electronic supplementary material:**

The online version of this article (10.1186/s12864-019-5463-1) contains supplementary material, which is available to authorized users.

## Background

**Fig. 1:**
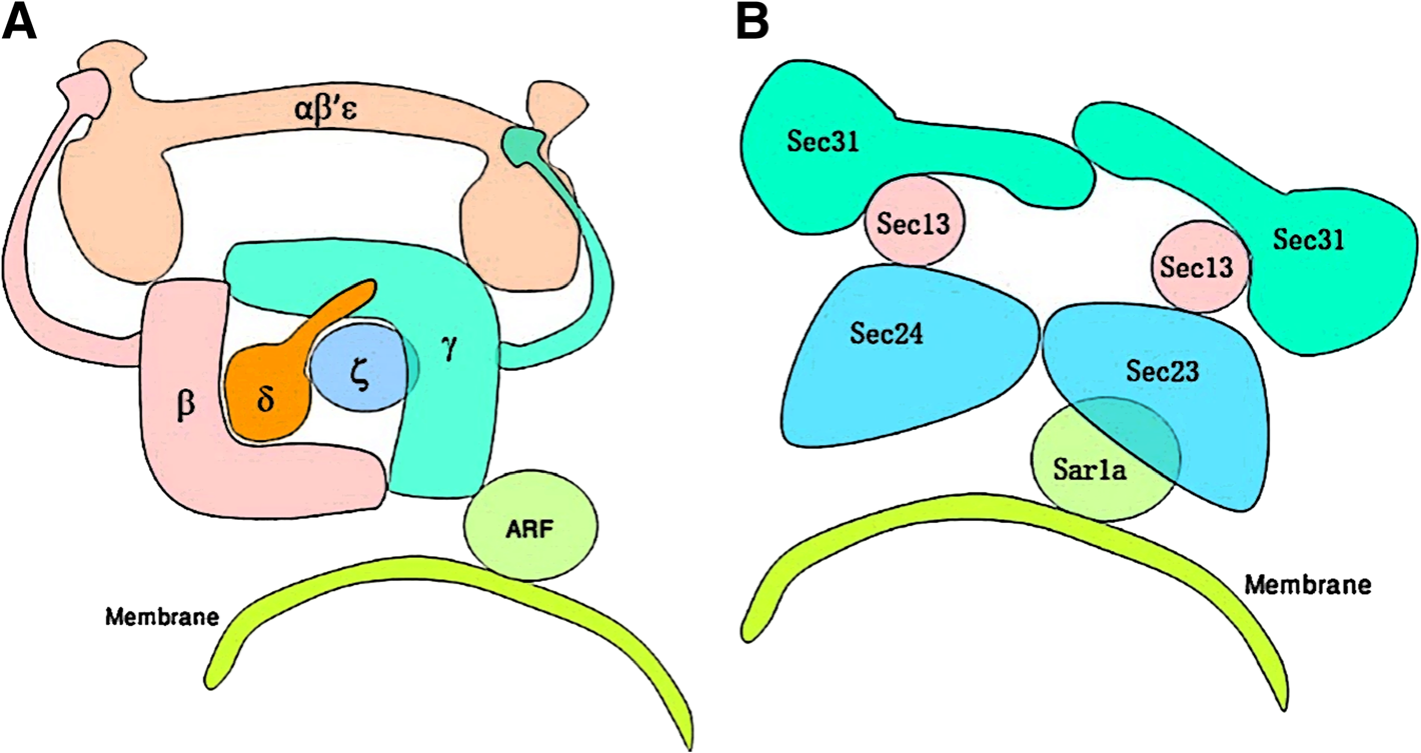
Coat protein composition of COPI and COPII vesicles. **a**. Cartoon of COPI structure showing a large coatomer subunit alpha, beta prime and epsilon subunit; delta; beta; gamma; and zeta subunit respectively. **b**. the COPII subunits consist of Sar1a tethering with membrane that interact with sec23/sec24 subunit, sec13 and sec31 subunits

The Apicomplexa includes a number of obligate intracellular parasites such as *Plasmodium, Cryptosporidium, Theileria, Eimeria and Neospora* that are of medical and agricultural significance [1]. This phylum is divided into five principal groups: Haemosporidia, Piroplasmorida, Coccidia, Gregarinasina, and Cryptosporidium [2]. Apicomplexan parasites use a conserved mechanism to invade the host cells, which is initiated by sequential secretion of proteins from apical organelles. The general feature of these parasites is the presence of a specialized collection of secretory organelles referred to as rhoptries, micronemes and dense granules [1]. All newly synthesized secretory proteins destined to the secretory and endosomal systems are trafficked to ER and subsequently from ER to Golgi before being delivered to their final destination in eukaryotic cells [3]. Several live imaging studies have established the cargo movement showing that proteins are exported from rough endoplasmic reticulum (RER) via specialized ER exit sites (ERES) into ER to Golgi intermediate compartments (ERGIC), then to Golgi complex and the trans-Golgi network (TGN) [4, 5]. ERES are represented by smooth projections of ER that are coated with COPII coat components and ERES generated COPII vesicles transport cargo further [4]. COPI coat proteins mainly mediate the retrograde transport pathway that selectively recycles and sorts proteins back to ERGIC, however its role in the transport of cargo to the next compartment is not established yet [4]. The COPII complex is composed of two heterodimers of Sec23/24p and Sec13/31p as well as a small GTPase, Sar1p [6]. Membrane binding of Sar1p-GTP recruits Sec23/24p heterodimer sub-complex that in turn recruits Sce13/31 heterodimer subcomplex of COPII results the deformation into buds and consequently to vesicles [7]. On the other hand, COPI is divided into two subcomplexes, one consisting of α, β’ and ε subunits; and another consisting of the β, δ, γ and ζ subunits. The heterotetramer of COPI (β, δ, γ and ζ-COP) resembles the clathrin adaptor AP1 (Adaptor Protein complex-1) and AP2 whereas the β´-COP is similar to clathrin and has been proposed to form a polygonal cage [8, 9]. The arrangements of the subunits of COPI and COPII are depicted in a schematic diagram (Fig. 1).

Golgi is a highly conserved and essential organelle in eukaryotes having varied structural shapes and numbers. Most mammalian cells possess the canonical structure of Golgi consisting of a series of flattened cisternal membranes, having multiple stacked structures referred as Golgi stacks that are arranged in the perinuclear area [10], associated with ERES. However, some single-cell eukaryotes like *Giardia* spp. and *Entamoeba histolytica* lack typical Golgi stacks [11]. The budding yeast *Saccharomyces cerevisiae* completely lacks a stacked Golgi; however, it is represented in the form of dispersed cisternae or isolated tubular networks. In a well-characterized apicomplexan parasite; *Toxoplasma gondii,* electron microscopy observation revealed that the parasite consists of a single Golgi located apical to the nucleus and is closely associated with a single ERES [12] as shown by three-dimensional reconstruction of serial EM sections [13]. The flagellated protozoan parasite *Trypanosoma brucei* has one ERES and a Golgi stack present adjacent to ERES between the nucleus and the flagellar pocket [14]. The biogenesis of Golgi apparatus both in *T. gondii* and *T. brucei* is associated with centrosomes/ basal bodies but duplicated by the different mechanisms [15]. Golgi stacks have not been observed in *Cryptosporidium* [16] and *Babesia* [17]*,* but are described in *Plasmodium falciparum* as an unstacked Golgi with single cisternae [18, 19]. In spite of having atypical Golgi, protein trafficking in Apicomplexans depends on vesicular carriers that fuse with distinct organelles to deliver proteins and lipids [20]. There have been limited studies with regards to the classical vesicle-mediated protein trafficking pathways in apicomplexan parasites. However, two consecutive *in-silico* studies have identified the AP complexes [21] and Epsin [22] in the Apicomplexans. Among the four AP complexes identified in Apicomplexan, only AP1 has been characterized in *P. falciparum* [23] and *T. gondii* [24] and shown to be associated with Golgi related protein sorting to Rhoptry, an invasive organelle. In this present study, we investigated apicomplexan genomes for the putative components of the COPI and COPII machinery. We performed comparative genomics and phylogenetic analyses to understand the evolutionary relationship of the coatomer subunits in the apicomplexan genome and their domain analyses for the functional significance.

## Methods

**Fig. 2:**
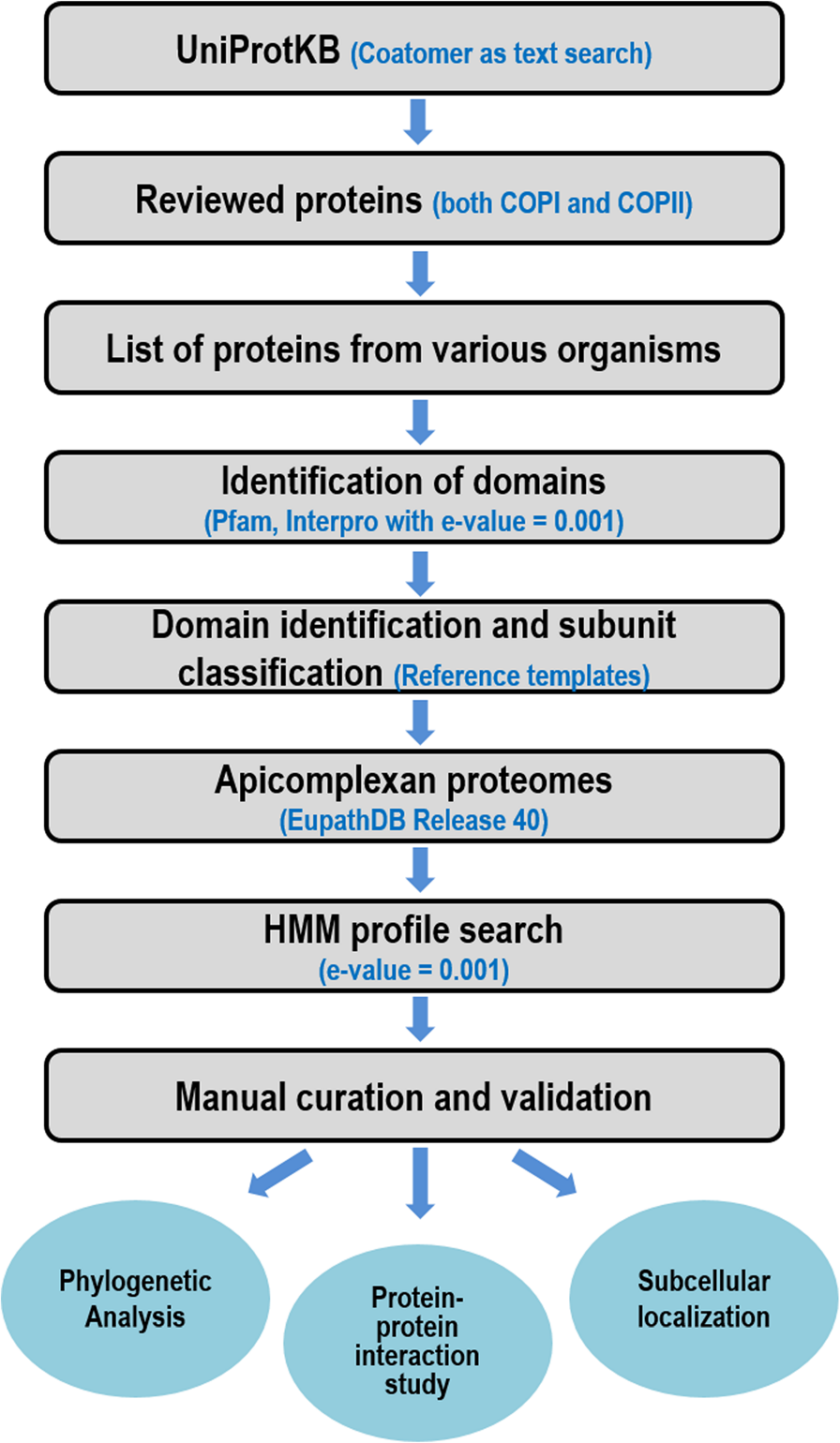
Flowchart of the methodology utilized to the identification of Coatomer subunits

The methodology utilized for identification and classification of Coatomer proteins and determination of their phylogenetic relationship has been described in the Fig. 2.

### Retrieval of COP sequences

COP sequences were retrieved by “coatomer” as a text search from the Uniprot database (updated on 25-05-2018). Only the proteins reviewed by Swiss-Prot were retained in order to remove false positives and were used as reference proteins for apicomplexans.

Also, the proteomes of six *Plasmodium* species {*Plasmodium falciparum* (3D7 strain)*, Plasmodium vivax* (strain Sal-1)*, Plasmodium berghei* (strain ANKA)*, Plasmodium chabaudii* (strain chabaudii)*, Plasmodium knowlesi* (strain H) *and Plasmodium yoelii* (strain yoelii 17x)} from PlasmoDB (http://plasmodb.org/plasmo/); *Eimeria tenella* (strain Houghton)*, Neospora caninum* (strain Liverpool) *and Toxoplasma gondii* (strain GT1) from ToxoDB (http://www.toxodb.org/toxo/); *Cryptosporidium parvum* (strain lowa II) from CryptoDB (http://cryptodb.org/cryptodb/); *Babesia bovis* (strain T2Bo)*, Theileria annulata* (strain ankara) and *Theileria parva* (strain Muguga) from PiroplasmaDB (http://piroplasmadb.org/piro/); and were used for the searches. Subunits of COPI and COPII of *Homo sapiens, Mus musculus, Arabidopsis thaliana* and *Saccharomyces cerevisiae* were also retrieved from the NCBI (http://www.ncbi.nlm.nih.gov/) database. The *Phytophthora sojae* was used as a outgroup for this study and the genome sequence was obtained from the Joint Genome Institute database (https://genome.jgi.doe.gov/Physo3/Physo3.home.html) [25].

### Identification and classification of COP proteins

HMM search was performed on all the 13 apicomplexan proteomes and on query proteins retrieved from Uniprot using HMMPfam [26] ver 2.3.2 with an e-value of 0.001 as cut off. This database has a large collection of protein families described by profile Hidden Markov Models, which are produced by multiple sequence alignments of the family member sequences [27]. In order to predict the coatomer proteins in apicomplexans, we have search the query proteins Pfam profiles in all the apicomplexan proteomes studied here. Those proteins that were mapped with the query pfam profiles were retained and checked manually for classification into COPI and COPII. For representation of domain architecture of COPI and COPII of the apicomplexan parasite in comparison to model organisms, HMMER 2.3.2 with an E-value of 0.001.

### Homology searching for validation

To validate homologous coatomer proteins, we used BLASTp similarity search. The similarity search was initially performed by using the default BLASTp parameter (version 2.2.28+, with Expectation value = 10, Maximum = 50) [28] on all of the above-mentioned spp. For the entire search, the *H. sapiens* COP homologues were used as initial queries against all the compared spp. Human proteins were selected because of their well-characterized domain structures and functional properties. If any of the homologs were not detected, we used related apicomplexan homologs especially from *P. falciparum* and *T. gondii*. We have also confirmed the annotation by domain analyses and consecutive search in the model organisms using NCBI BLASTp.

### Phylogenetic analysis

MSA was performed for the selected COPI and COPII protein sequences using MUSCLE integrated into MEGA7 [29] with Gap opening = − 2.90, Gap extend = 1.20, Hydrophobicity Method = 1.20 and Iterations = 16. The tree was constructed using Maximum likelihood method, Phylogeny test = Bootstrap method, No of Bootstrap Replications = 500, Substitution model = Jones Taylor Thorton, Rates and Patters = Uniform rates, Gaps/Missing Data = Partial deletion and Sites Coverage cut off = 95.

### The prediction of subcellular localization of COPI and COPII subunits

To further predict localization of coatomer proteins we have performed subcellular localization study using CELLO (http://cello.life.nctu.edu.tw/) against eukaryotes as targets.

### Protein-protein interaction analyses

The interaction of the COPI and COPII protein components were analyzed using STRING. STRING uses neighbourhood, gene fusion, co-expression, co-occurrence, homology, text mining, database information and experiments to list the interacting partners using the scoring method. We have only retrieved the interacting partners with strong binding having the first shell of interaction and the second shell of interaction. The further interaction was prohibited to reduce the complication of the data. The co-expression data for the proteins having the first shell of interaction within COPI and COPII components have been downloaded from STRING. The co-expression has been represented for *Plasmodium falciparum* and also in other organisms to compare their functional clustering. We have checked the transcriptomics data (microarray-based) of the different developmental stages of the 3D7 strain of *P. falciparum.* The expression data of an oligonucleotide array of *P. falciparum* was downloaded from PlasmoDB [30].

## Results

### Identification of COPI and COPII subunits in apicomplexan genome

Uniprot search resulted in identification of 54 and 150 reviewed proteins, in COPI and COPII respectively. There were 11 different Pfam domains traced to be distributed in COPI and COPII proteins (Table 1 and Table 2) of various model organisms that were used as query Pfam profiles.

**Table 1:**
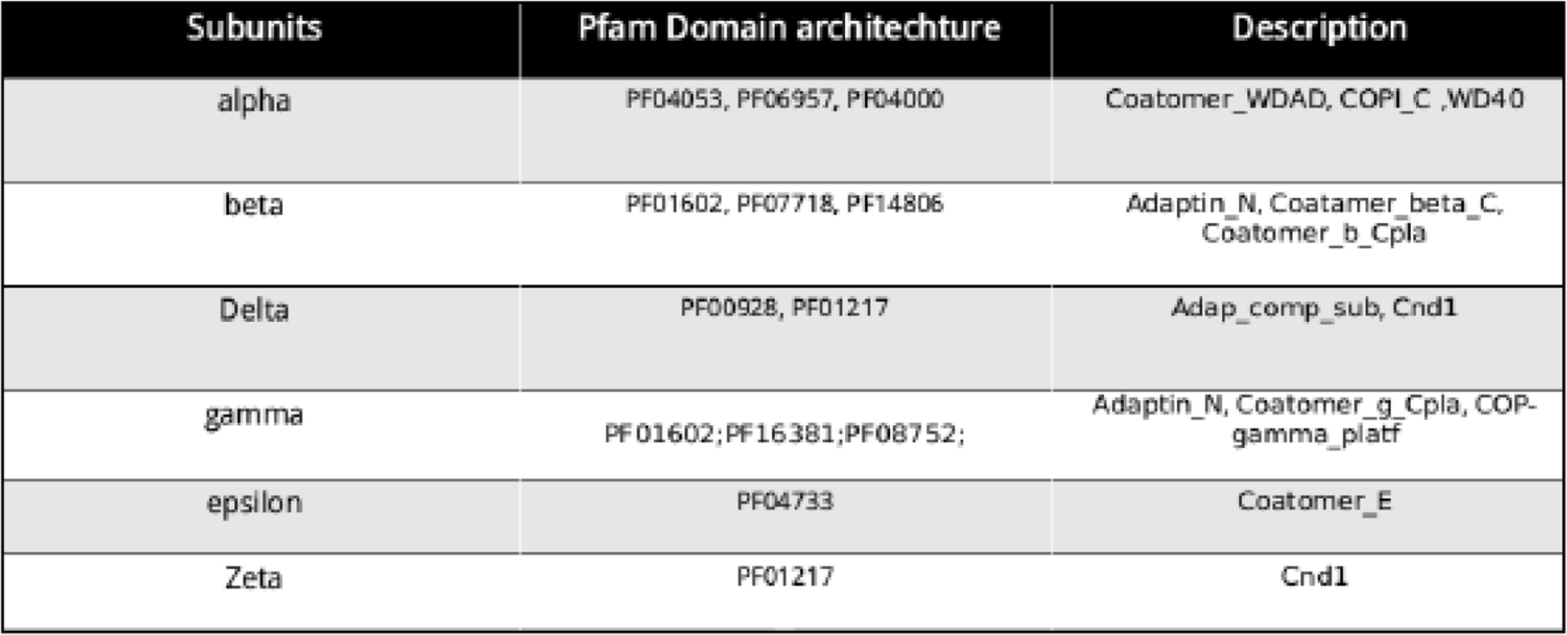
Pfam profiles of COPI subunits of model organisms

**Table 2:**
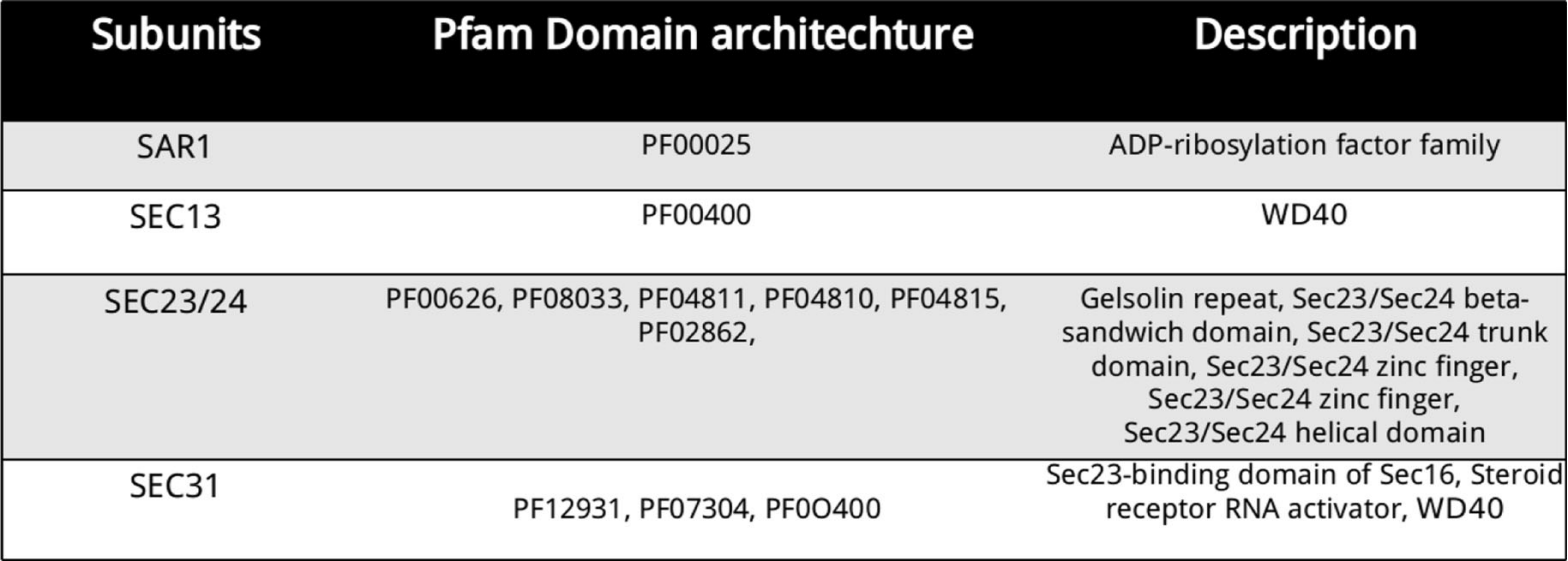
Pfam profiles of COPII subunits of model organisms

**Fig. 3:**
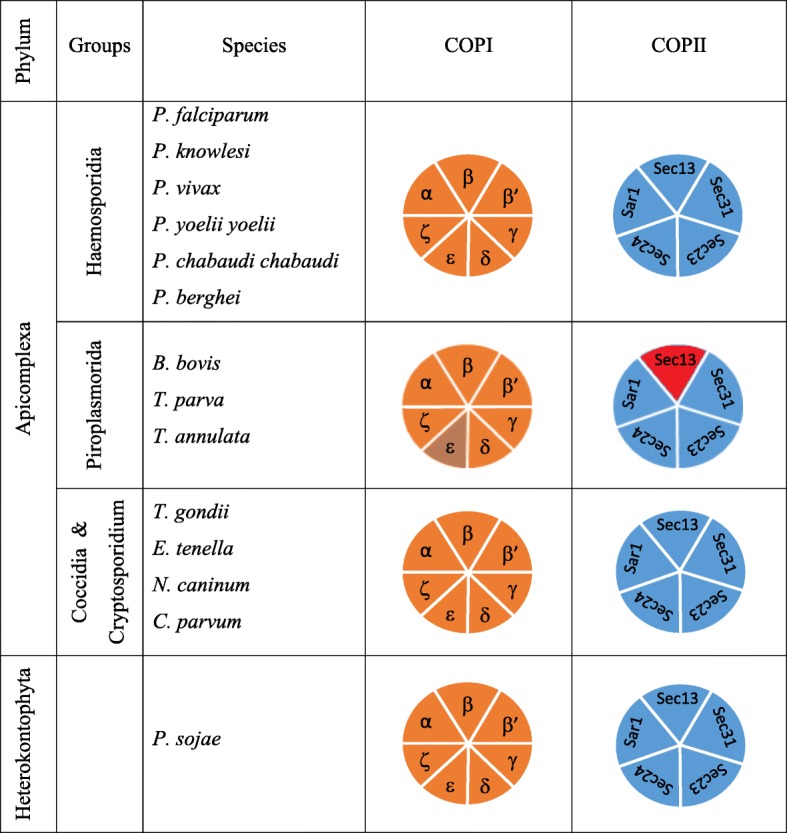
Plot representation of the COPI and COPII subunits in the apicomplexa. The blocks with contrasting color represent absence of subunits

COP II coat was first identified in the yeast *S. cerevisiae* and is composed of two heterodimers of Sec23/24p and Sec13/31p as well as a small GTPase, Sar1p [6]. Most of these components are conserved in eukaryotes and are essential for cell viability [31]. Like-wise COPI complex consists of seven coatomer proteins- α,- β,- β’,- λ,-δ,-ε,-ς that with ARF-family G protein form COPI coated vesicles [32]. We screened the proteomes of apicomplexan parasites using in silico computational approaches and identified all the subunits of COPI and COPII complexes. As shown in Fig. 1, we identified all the subunits of COPI complex in most of the Apicomplexan organisms with the exception of absence of epsilon subunit in *B. bovis*, *T. annulata* and *T. parva* genomes. Likewise, we identified all the subunits of COPII complex except sec13 subunit being absent in *B. bovis*, *T. annulata* and *T. parva* genomes (Fig. 3)*.* This lack of a single subunit in these three organisms is indicative of a secondary loss of protein in these three genomes similar to what has been proposed in case of adaptin complexes of apicomplexan parasites [21].

The gene names of apicomplexan COPI and COPII homologs, their gene IDs along with their location in the respective position of genes in the chromosome and relative gene and protein characteristics are shown in Additional file 1: Tables S1-S13.

### The phylogenetic analyses of COPI and COPII subunits in apicomplexans

**Fig. 4:**
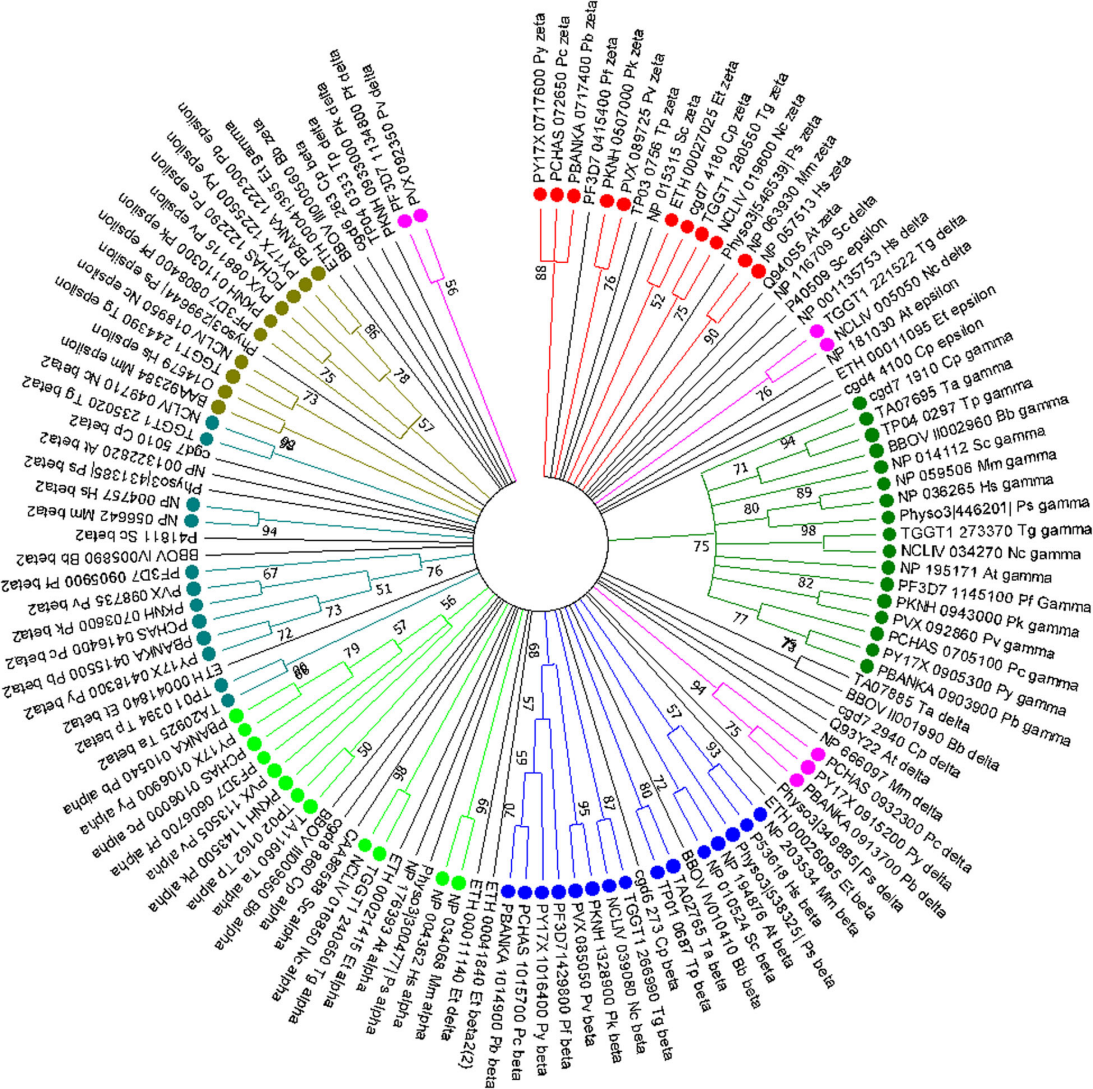
Circular cladogram of COPI protein complex. Molecular Phylogenetic analysis by Maximum Likelihood method. The evolutionary history was inferred by using the Maximum Likelihood method based on the JTT matrix-based model [44]. The bootstrap consensus tree inferred from 500 replicates [45] is taken to represent the evolutionary history of the taxa analyzed [45]. Branches corresponding to partitions reproduced in less than 50% bootstrap replicates are collapsed. Initial tree(s) for the heuristic search were obtained automatically by applying Neighbor-Join and BioNJ algorithms to a matrix of pairwise distances estimated using a JTT model, and then selecting the topology with superior log likelihood value. The analysis involved 124 amino acid sequences. All positions containing gaps and missing data were eliminated. There were a total of 20 positions in the final dataset. Evolutionary analyses were conducted in MEGA7 [29]. Beta’ subunits were written as Beta 2 in the tree. Hs, *Homo sapiens*; Mm, *Mus musculus*; At, *Arabidopsis thaliana*; Sc, *Saccharomyces cerevisiae*; Pf, *Plasmodium falciparum*; Pk, *Plasmodium knowlesi*; Pv, *Plasmodium vivax*; Py, *Plasmodium yoelii yoelii*; Pc, *Plasmodium chabaudi chabaudi*; Pb, *Plasmodium berghei*; Tg, *Toxoplasma gondii*; Cp, *Cryptosporidium parvum*; Bb, *Babesia bovis*; Ta, *Theileria annulata*; Nc, *Neospora caninum*; Et, *Eimeria tenella*; Tp, *Theileria parva*; Ps, *Phytophthora sojae*

**Fig. 5:**
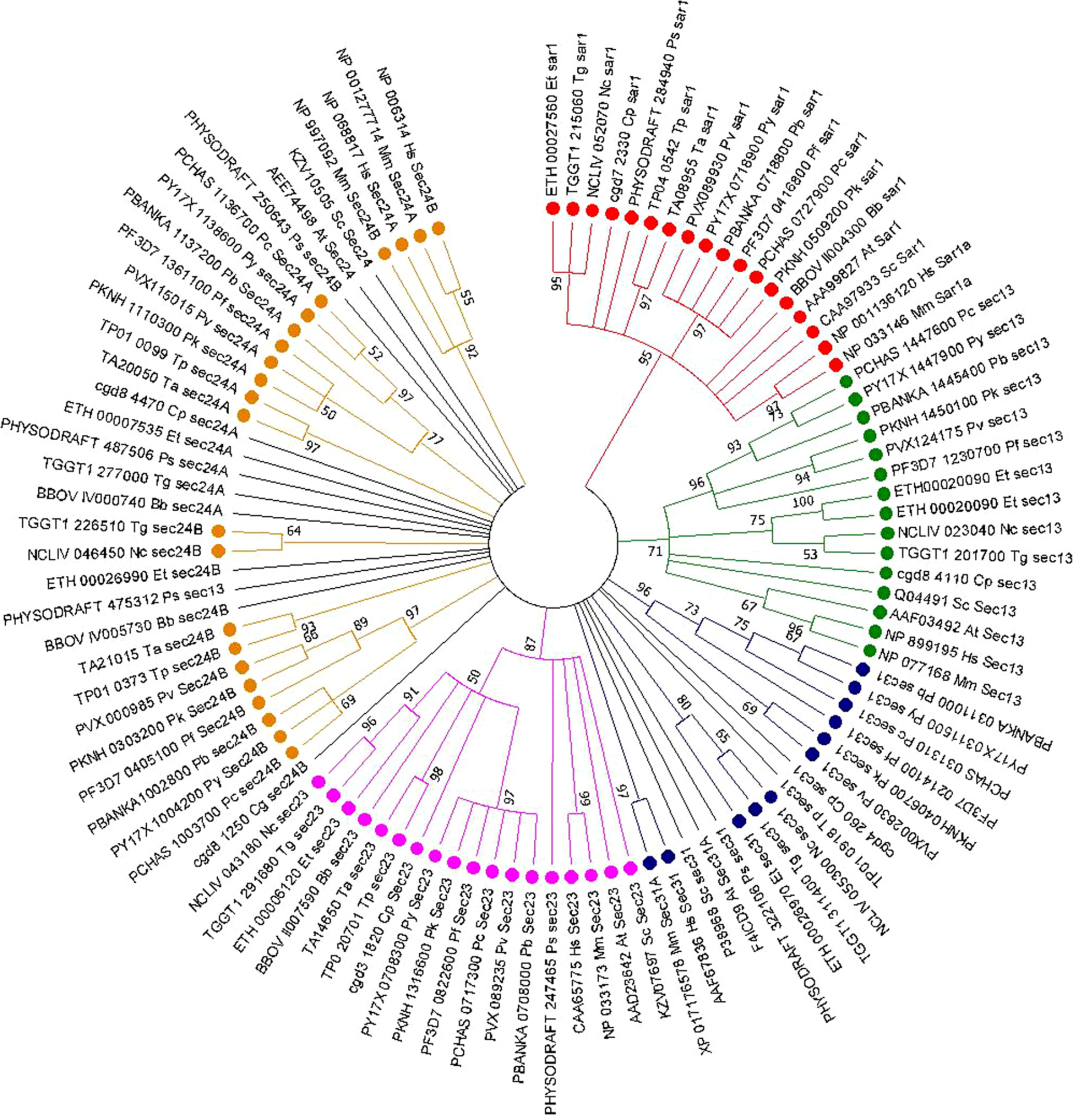
Circular cladogram of COPII protein complex. Molecular Phylogenetic analysis by Maximum Likelihood method. The evolutionary history was inferred by using the Maximum Likelihood method based on the JTT matrix-based model [44]. The bootstrap consensus tree inferred from 500 replicates [45] is taken to represent the evolutionary history of the taxa analyzed [45]. Branches corresponding to partitions reproduced in less than 50% bootstrap replicates are collapsed. Initial tree(s) for the heuristic search were obtained automatically by applying Neighbor-Join and BioNJ algorithms to a matrix of pairwise distances estimated using a JTT model, and then selecting the topology with superior log likelihood value. The analysis involved 101 amino acid sequences. All positions containing gaps and missing data were eliminated. There were a total of 20 positions in the final dataset. Evolutionary analyses were conducted in MEGA7 [29]. Hs, *Homo sapiens*; Mm, *Mus musculus*; At, *Arabidopsis thaliana*; Sc, *Saccharomyces cerevisiae*; Pf, *Plasmodium falciparum*; Pk, *Plasmodium knowlesi*; Pv, *Plasmodium vivax*; Py, *Plasmodium yoelii yoelii*; Pc, *Plasmodium chabaudi chabaudi*; Pb, *Plasmodium berghei*; Tg, *Toxoplasma gondii*; Cp, *Cryptosporidium parvum*; Bb, *Babesia bovis*; Ta, *Theileria annulata*; Nc, *Neospora caninum*; Et, *Eimeria tenella*; Tp, *Theileria parva*; Ps, *Phytophthora sojae*

Sequence analyses revealed three distinct clades of COPI and COPII complexes in apicomplexan genomes. All the COPI and COPII genes in the genus *Plasmodium* show a high degree of similarity, thus form separate clades for each set of genes. The epsilon and sec13 subunit are absent in *B. bovis, T. annulata,* and *T. parva* genome. The other COPI subunits of *T. annulata,* and *T. parva* cluster in a clade indicating a significant sequence similarity, whereas *B. bovis* sequences showed diversity. The COPI genes of *T. gondii* and *N. caninum* show high similarity and form a clade, whereas the COPI genes of *E. tenella, P. sojae,* and *C. parvum* showed a high degree of diversity. The COPII subunits of *T. annulata,* and *T. parva* cluster together, likewise *T. gondii, E. tenella,* and *N. caninum* COPII subunits are also in a separate clade showing a high degree of sequence similarities. However, *B. bovis, P. sojae,* and *C. parvum* COPII genes have diverse sequences than other apicomplexan genomes. The sec24A subunit of piroplasmorida, coccidia and cryptosporidium are very much diverse and located in a scattered way in the tree. In the case of delta subunit the apicomplexan homologs also showed the diversity and are represent into three separate clades. The circular cladogram for COPI (Fig. 4) and COPII complexes (Fig. 5) illustrate the phylogenetic relationship between the various subunits of COPI & COPII amongst some of the well-characterized organisms such as Humans, Mouse, *Arabidopsis,* and *Saccharomyces.*

### Annotation of the COPI and COPII subunits of Apicomplexa

**Fig. 6:**
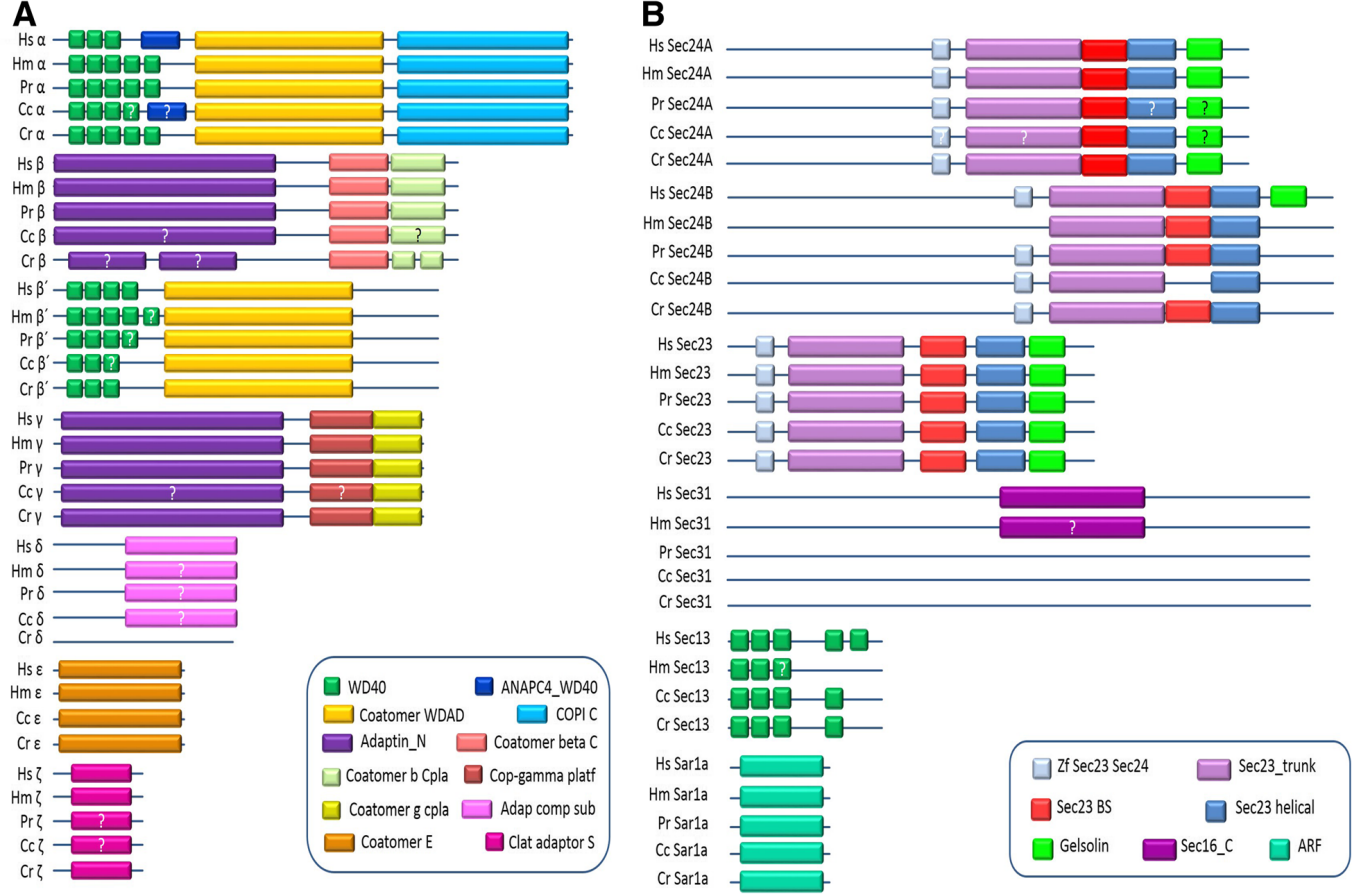
The comparison of domains of **a**. COPI and **b**. COPII in Haemosporidia (Hm), Piroplasmorida (Pr), Coccidia (Cc) and Cryptosporidium (Cr) with that of the *Homo sapiens* (Hs). The Haemosporidia includes all the *Plasmodium* species, the Piroplasmorida includes the *B. bovis, T. parva* and *T. annulata*, the Coccidia includes *T. gondii, E. tenella* and *N. caninum* and the Cryptosporidium includes the species *C. parvum*. The sign “?” means that diversity is there, either by absence or differ by numbers of domains. Note: The protein length and the position of domains has not been compared

A number of subunits of COPI and COPII complexes have erroneous annotations in the apicomplexan genome database, especially all the Beta’ subunits have been annotated as coatomer of subunits beta, putative. In the present analysis, we show that the beta and beta’ subunits have major differences in their domain organization. Human COPI beta subunit consists of an N-terminal “adaptin N” domain followed by a “Coatomer b Cpla” and a “Coatomer beta C” domain. Whereas, beta’ subunit of apicomplexan parasites contains a C-terminal “Coatomer_WDAD” domain preceded by approximately four consecutive WD40 domain that is totally different from that of the beta subunit (Fig. 6a). The updated annotations of the respective subunits are shown in Additional file 1: Table S1-S13.

Though the epsilon subunits of COPI are absent from the genome of *B. bovis*, *T. annulata* and *T. parva* the respective sec13 subunits of COPII has an ambiguous organization. Sec13 is a WD40 domain containing protein. Since many apicomplexan proteins possess WD40 domain, it was difficult to search for an actual sec13 homologue in these organisms. We selected some of the candidate proteins having WD40 domain found from BLAST search and perform multiple sequence alignment with the human homologs. The search with sec13 of *H. sapiens* and *P. falciparum* revealed some candidate proteins such as TA17990 of *T. annulata,* BBOV_III003670, and BBOV_III003640 of *B. bovis* and TP03_0734 of *T. parva*. However, phylogenetic analyses suggested that they are very distantly related to sec13 (Additional file 2: Figure S1). Moreover, domain analyses showed that candidate proteins of *Babesia* do not have WD40 domain and TP03_0734 contains a single WD40 domain, whereas TA17990 contains 5 (five) WD40 domains along with an MPC domain. Further studies in particularly protein-protein interaction studies are warranted to establish the proper identity and annotation of the sec13 protein in these organisms. Finally, we searched the piroplasmorida genome by tBLASTN and tBLASTx to find the distantly related sec13 homolog. However, these analyses confirmed that sec13 is clearly absent in the piroplasmorida genome.

### The domain architecture of apicomplexan COPI and COPII

#### COPI

The Domain architecture of Apicomplexan COPI subunits are depicted in Fig. 6a and described in Additional file 1: Table S14. The general domain architecture of COPI alpha subunits of apicomplexan parasites is similar to that of human homolog except for WD40 domains. The numbers of WD40 domain varies among the parasites. In coccidians, COPI alpha subunits possess 3 to 6 WD domains, however, five WD40 domains are present in other apicomplexans. The COPI C domain at C-terminal is absent in *P. knowlesi* and one single domain called ANAPC4_WD40 is present in *N. caninum* similar to that of the *H. sapiens*.

The domain architecture of COPI beta subunits is also conserved except for the absence of “Adaptin N” domain in *E. tenella*. A single domain of “Coatomer b Cpla” is present throughout the apicomplexan and humans, with an exception being *T. gondii* that has two Coatomer b Cpla domains. However, there is a diversity in the domain architecture in beta’ subunits especially on the number(s) of WD40 domains. Haemosporidia, Piroplasmorida, and Coccidia COPI beta subunits contain 3–4, 2–4 and 2–3 WD40 domains respectively at their N-termini. Gamma subunit is very much conserved across the apicomplexan with the exception of *E. tenella* COPI Gamma subunit that lacks the “Adaptin N” domain along with a “Cop-gamma platf” domain. Interestingly, only the epsilon subunit of COPI is fully conserved among all the apicomplexan. The delta subunits are diverse and most of the parasites lack the conserved domain found in human homolog. The conserved “Clat adaptor S” domain of zeta subunit is absent in *E. tenella* and *T. parva*. On the whole, it seems that *E. tenella* has the most diverse COPI subunit in terms of the domain architecture. However, *T. parva, T. annulata,* and *B. bovis* COPI subunits also show some diversity.

#### COPII

The Domain architecture of Apicomplexan COPII subunits are depicted in Fig. 6b and described in Additional file 1: Table S14. The prominent subunits that help to tether the COPII complex with the membrane are sar1 and sec23. These domains are very much conserved in terms of architecture in apicomplexan. The sar1a subunit of COPII consists of Arf domain. All the Sec23, Sec24A, and Sec24B contain similar domains architecture having five different domains namely Zf Sec23 Sec24, Sec23 trunk, Sec23 Beta-Sandwich (Sec23 BS), Sec23 helical and Gelsolin. Most of the apicomplexan parasites have diverse domain architecture for Sec24A and Sec24B subunits when compared with human homolog. The sec24A of Haemosporidia and *Cryptosporidium* are conserved but in the subphylum coccidia, *E. tenella* sec24A subunit possess two “sec23 trunk” domain and *N. caninum* lacks the “Zf sec23 sec24” domain. The “Gelsolin” domain of Sec24A is absent in both *E. tenella,* and *N. caninum.* In piroplasmorida, *T. parva and T. annulata* lack both the “Sec23 helical” and “Gelsolin” domains.

Sec24B of all the apicomplexan are diverse when compared with Human Sec24B homolog; they lack “Gelsolin” domain. Haemosporidia Sec24B subunit lacks the “Zf sec23 sec24” domain, whereas *E. tenella* and *N. caninum* Sec24B subunit lack the “Sec23BS” domain. The Human COPII Sec13 subunit possess five WD40 domains. However, the Sec13 subunit of Haemosporidia*,* Piroplasmorida, and Coccidia contain 2–3, 1–6 and 3–4 WD40 domains respectively. Sec31 of human comprises of “Sec16_C” domain that is present in most of the Haemosporidia such as *P. falciparum, P. knowlesi, P. yoelii, P. chabaudi, P. berghei* and *P. sojae* but missing in the remaining members of apicomplexan.

### The prediction of subcellular localization of COPI and COPII subunits

Most of the COPI subunit has been found to be localized in Nucleus, Cytoplasm, Plasma Membrane and Golgi. In case of COPII subunits the localization was found to be in the Nucleus and Plasma membrane (Additional file 3: Table S18 and Additional file 4: Figure S6).

### The interaction networks for COPI and COPII subunits

To illustrate the protein-protein interactions among the subunits of COPs in apicomplexan parasites, we selected *P. falciparum* as a model organism due to the availability of the interaction data in the STRING database, as it has been well studied in *Plasmodium* among all the apicomplexan parasites. Among the first and second shell of interaction, we found 22 interacting proteins of COPI having avg. local clustering coefficient 0.790 and 23 interacting proteins of COPII having avg. local clustering coefficient 0.894. The interaction of the proteins of COPI and COPII and their co-expression in *P. falciparum* as well as in other organisms are shown in Additional file 5: Figure S2 and Additional file 6: Figure S3 and described in Additional file 1: Table S15 and Additional file 7: Table S16, Table S17. The COPI interaction data shows that the Alpha (PFF0330w), Beta (PF14_0277), Beta’ (PFI0290c), Gamma (PF11_0463), Delta (PF11_0359), Epsilon (MAL8P1.121) and Zeta (PFD0745c) subunit of COPI complex are co-expressed in *P. falciparum* and there are prediction based evidences that they interact with each other as depicted in Fig. 1a. The experimentally determined interaction values are > 0.9, the specific interactions and co-expression values are shown in the bold font in Additional file 7: Table S16. The interaction data of COPII shows that the Sar1, Sec23, Sec24A (PF13_0324), Sec24B (PFD0250c), Sec13 (PFL1480w) and Sec31p subunits of COPII complex are co-expressed as well and interact consequently. The experimentally determined interaction values are > 0.8 except for two interactions i. Sec24A and Sec13 and ii. Sar1 and Sec23 that shows moderate interaction (> 0.6). The specific interactions and co-expression values for COPII subunits are shown as bold in Additional file 7: Table S17 that provides prediction based evidences for the formation of COPII complex as depicted in Fig. 1b. To check the expression level in the *P. falciparum*, we have also analyzed the microarray data of the different stages of the parasite. These proteins are expressed in all the stages of the life cycle of the parasites and the expression pattern is similar that has also been shown by a heat map (Additional file 8: Figure S4, Additional file 9: Figure S5 and Additional file 10: Table S19).

In summary results presented here show that all the components of COPI and COPII complex are conserved among apicomplexan parasites, thereby suggesting the existence of a protein trafficking machinery similar to the one present in higher eukaryotes.

## Discussion

The availability of the genomic sequences of the apicomplexan parasites has provided a huge sequence database that on analysis can provide a clear picture of the evolutionary relations of these parasites with higher eukaryotes. Most of the parasites in this phylum are obligate intracellular parasites that live in multiple hosts for their survival. They face a huge selection pressure to confront the host immune system and therefore have developed diverse protein trafficking system to carry out their inhabitation inside the host cell. However, the existence of a rudimentary Golgi or its absence in these parasites points towards the divergence in their classical vesicle-mediated pathway. These parasites possess a wide range of protein trafficking systems based on a number of studies mainly performed in *P. falciparum,* a highly studied parasite of this phylum. In *P. falciparum,* a lot of proteins are distributed to the unique subcellular organelles and are also exported to the host red blood cell [33]. These eukaryotic cells usually comprise functional compartmentalization in the form of endomembrane system. Electron microscopy studies have shown an ER in *P. falciparum* that proliferates extensively throughout the parasite cytoplasm [34] and is continuous with the nuclear envelop [35]. The parasite also possesses a rudimentary Golgi-like complex, which appears as a collection of one or a few tubular or flat cisternae surrounded by vesicles of various sizes [18, 35, 36]. Cargo proteins can be packaged and vesicles can be formed from both of these organelles to target proteins in a highly regulated way [37].

In the present study, we performed a comparative genomic and phylogenetic analyses to delineate the COPI and COPII complexes of apicomplexan parasites. Our analysis revealed that COPI epsilon subunit and COPII sec13 subunit are absent in the genome of *B. bovis, T. parva, and T. annulata*. A similar type of secondary loss of adaptin complex subunits had been reported by Nevin et al. [21], which is the component of Endosomal Sorting Complexes Required for Transport (ESCRT) proteins form the machinery necessary for the material to enter the multivesicular body, one of the key organelles of the endocytic pathway. Comparative genomics revealed that while ESCRT complexes III and III-associated were retained, the ESCRT I and II complexes were missing in many of the Apicomplexa sampled so far [38].

There may be several reasons that might contribute to the lack of the subunit or difficulty in their identification. One such reason is the diversity of the sequences of genes and incompleteness of the genome database, although we attempted to use not only the query sequence from human or mouse homologs but also the homologs from other apicomplexans (e.g. *P. falciparum, T. gondii*) to search for the unidentified homologs to reduce the divergence effect in the present analysis. So, we are relatively confident to predict the absence of epsilon subunit in the respective genome database.

Though most of the conserved domains present in the apicomplexan COPI and COPII subunits, the phylogenetic analyses showed that Apicomplexan Coatomer subunits are diverse than the homologs in the model organisms. The Haemosporidia COPI and COPII are conserved but the *B. bovis, E. tenella, C. parvum,* and *P. sojae* coatomer subunits are much more diverse than the other apicomplexan homologs. The phylogenetic and domain analyses showed that the delta subunit of COPI and Sec24A subunit of COPII have a high degree of diversity as compared to other subunits. We have found three paralogs in the apicomplexan parasite (*C. parvum* beta subunit, *E. tenella* beta’ subunit and *T. parva* delta subunit) (Additional file 1: Table S2, Table S3, and Table S5). In most of the cases, these proteins were smaller in size and located adjacent in the genome separated by smaller sequence. This might be a result of poor sequencing or addition of stop codon inside a full gene.

The protein-protein interaction data from STRING shows that the COPI and COPII subunits interact with each other and co-expressed. In COPI interaction data, two important regulatory proteins have been identified such as GTPase activating protein (also known as GAP, PF08_0120) and ER lumen protein retaining receptor 1 (alternatively known as ERD2, MAL13P1.163.1). The luminal KDEL ligand binding induces oligomerization of ERD2 and recruitment of ARF-GAP into the Golgi, finally ERD2/ARF-GAP complex interacts with membrane-bound ARF [39]. ARF-GAP can directly interact with beta and gamma subunits of COPI and regulate the formation of COPI coat in the membrane curvature [4]. Due to the interaction of beta subunits of adaptin complex with coatomer beta, beta’, gamma and epsilon subunits in the first shell of interaction, we found the other subunits of Adaptor protein complexes (AP-1, AP-2, AP-3 and AP-4) along with clathrin heavy chain in the second shell of interaction. However, this type of direct interactions between Coatomer and Adaptin has not been documented yet. As ARF-GAP can also induce the recruitment of AP-complexes in the Golgi [23], it might be acting as a hub for COP and adaptin complexes.

In case of COPII complex, most of the subunits interact tightly while two moderate interactions were found. Sec24A has less interaction with Sec13 than Sec24B indicating that the major COPII complex was formed with Sec24B rather than Sec24A in *P. falciparum* and the transient interaction between Sar1 and Sec23 might be functionally significant for its ability to regulate vesicle formation. Sar1 is a special type of protein that mediates membrane remodeling with the help of GTP hydrolysis [40]. The GTP bound form of Sar1 embed into the membrane and recruits the Sec23/Sec24 complex to form “pre-budding” complex. Finally the “pre-budding” complex recruits the Sec13/Sec31 to nucleate the polymerization of multiple Sec13/Sec31 assembly units to form cage [41]. So, for membrane deformation the specific interaction between the subunits are essential. ERD2, Rab1 and Rab2 interaction suggests that they might be acting as regulatory proteins for COPII complexes. Some fusogenic proteins like NSF, SNARE, Syntaxins and Syntaxin binding proteins are found to be interacted in the first and second shell of interaction with COPII complex. These fusogenic proteins are mainly associated with vesicle fusion and also implicated to regulate vesicle trafficking by COPII [42] and COPI [43].

## Conclusions

The present study identifies the components of COPI and COPII complexes that are involved in protein secretion among the apicomplexan parasites. The phylogenetic analyses of these coatomer proteins highlight the diversity of these proteins with their homologs among the apicomplexan parasites and higher eukaryotes. Detailed interactome analyses indicate their assembly at subunit level and also with some regulatory proteins. The stringent annotation and comprehensive listings of apicomplexan COPI and COPII proteins in this study will be helpful to illustrate the protein secretory pathway in the Apicomplexans. The functional validation of these proteins in the respective parasites may lead to disclosure of specific role of these proteins in organelle biogenesis, nutrient acquisition and parasitic invasive mechanism.

## Additional files

Additional file 1: **Table S1.** The coatomer alpha homologues of apicomplexan parasites with their characteristics. **Table S2.** The coatomer Beta homologues of apicomplexan parasite. **Table S3.** The coatomer Beta’ homologues of apicomplexan parasites. **Table S4.** The coatomer Gamma homologues of apicomplexan parasites. **Table S5.** The coatomer Delta homologues of apicomlexan parasites. **Table S6.** The coatomer Epsilon homologues of apicomplexan parasites. **Table S7.** The coatomer Zeta homologues of apicomplexan parasites. **Table S8.** Coatomer sec13 homologues of apicomplexan parasites. **Table S9.** The Coatomer sec31 Homologues of apicomplexan parasites. **Table S10.** The coatomer sec23 homologues of apicomplexan parasites. **Table S11.** The coatomer sec24A homologues of apicomplexan parasites. **Table S12.** The coatomer sec24B homologues of apicomplexan parasites. **Table S13.** The Coatomer Sar1a homologues of apicomplexan parasites. **Table S14.** The domain architecture of the COPI and COPII subunits of apicomplexan parasites in comparison to that of the human homologs. The number of domain(s) and their locations are mentioned for the corresponding proteins. **Table S15.** The list of proteins identified to have interaction and co-expression for COPI and COPII. (DOCX 151 kb)

Additional file 2: **Figure S1.** The phylogenetic tree showing the relation between the *T. annulata, T. parva* and *B. bovis* putative sec13 proteins with other apicomplexan and higher order organisms. (TIF 1427 kb)

Additional file 3: **Table S18.** Subcellular localization of the COPI and COPII proteins. (XLSX 42 kb)

Additional file 4: **Figure S6.** Subcellular localization of the COPI (A) and COPII (B) proteins. (PDF 230 kb)

Additional file 5: **Figure S2.** Protein-protein interaction data of COPI retrieved from String. A. The first shell of interaction with COPI components are shown by colored nodes and the second shell of interaction are shown by white nodes. B. The proteins that are co-expressed in *Plasmodium falciparum* has been shown in first graph and the co-expression in other organisms are shown in second graph. The pink color edges are indicating experimentally proved interactions. (PDF 578 kb)

Additional file 6: **Figure S3.** Protein-protein interaction data of COPII retrieved from String. A. The first shell of interaction with COPII components are shown by colored nodes and the second shell of interaction are shown by white nodes. B. The proteins that are co-expressed in *Plasmodium falciparum* has been shown in first graph and the co-expression in other organisms are shown in second graph. The pink color edges are indicating experimentally proved interactions. (PDF 533 kb)

Additional file 7: **Table S16.** The scores of interaction and co-expression among the COPI subunits and other interacting partners resulted from STRING. The interaction between the subunits of COPI supported by literature are shown as bold. **Table S17.** The scores of interaction and co-expression among the COPII subunits and other interacting partners resulted from STRING. The interaction between the subunits of COPII supported by literature are shown as bold. (XLSX 24 kb)

Additional file 8: **Figure S4.** The gene expression of COPI and COPII in different stages of Malaria parasite. The X-axis indicating different stages of malaria parasite and the Y-axis shows the normalized RMA value (log base 2). (PDF 243 kb)

Additional file 9: **Figure S5.** The heat map showing gene expression profiles of COPI and COPII in different stages of Malaria parasite. (PDF 427 kb)

Additional file 10: **Table S19.** The Expression of COPI and COPII subunits in different stages of *Plasmodium falciparum*. (XLSX 63 kb)

## Abbreviations

ARF: ADP-ribosylation factor
ARF-GAP: ADP-ribosylation factor-GTPase-activating *protein*
EM: Electron microscopy
GTPase: Guanosine triphosphatase
HMM: Hidden Markov Model
MPC: Motor *protein* complex
MUSCLE: Multiple sequence comparison by log expectation
NSF: N-Ethylmaleimide-Sensitive Factor
Rab: Ras-related in brain
SNARE: Soluble NSF (N-Ethylmaleimide-Sensitive Factor) Attachment *Protein* Receptor
