# Supplementary material for: A genome-wide analysis of coatomer protein (COP) subunits of apicomplexan parasites and their evolutionary relationships
Source: BMC Genomics. 2019 Jan 31;20:98. doi: 10.1186/s12864-019-5463-1 (PMC6357402; doi:10.1186/s12864-019-5463-1)
Supplement: Supplementary file 1 — Table S1. The coatomer alpha homologues of apicomplexan parasites with their characteristics. Table S2. The coatomer Beta homologues of apicomplexan parasite. Table S3. The coatomer Beta’ homologues of apicomplexan parasites. Table S4. The coatomer Gamma homologues of apicomplexan parasites. Table S5. The coatomer Delta homologues of apicomlexan parasites. Table S6. The coatomer Epsilon homologues of apicomplexan parasites. Table S7. The coatomer Zeta homologues of apicomplexan parasites. Table S8. Coatomer sec13 homologues of apicomplexan parasites. Table S9. The Coatomer sec31 Homologues of apicomplexan parasites. Table S10. The coatomer sec23 homologues of apicomplexan parasites. Table S11. The coatomer sec24A homologues of apicomplexan parasites. Table S12. The coatomer sec24B homologues of apicomplexan parasites. Table S13. The Coatomer Sar1a homologues of apicomplexan parasites. Table S14. The domain architecture of the COPI and COPII subunits of apicomplexan parasites in comparison to that of the human homologs. The number of domain(s) and their locations are mentioned for the corresponding proteins. Table S15. The list of proteins identified to have interaction and co-expression for COPI and COPII. (DOCX 151 kb) [file 12864_2019_5463_MOESM1_ESM.docx]

**Table S1:** The coatomer alpha homologues of apicomplexan parasites with their characteristics.

| Protein | Gene ID | Gene Name | Organism | Location  (chr.no./position) | Size (protein)aa/KDa | Exon (no.) | Intron (no.) |
| --- | --- | --- | --- | --- | --- | --- | --- |
| Pf Alpha | PF3D7_0606700 | coatomer alpha subunit, putative | *Plasmodium falciparum* | 6/ 282,049-287,017 | 1512/176.95 | 4 | 3 |
| Pk Alpha | PKNH_1143500 | coatomer alpha subunit, putative | *Plasmodium knowlesi* | 11/ 2022333 – 2027014 | 1365/ 156.85894 | 5 | 4 |
| Pv Alpha | PVX_113505 | coatomer alpha subunit, putative | *Plasmodium vivax* | 11/1,747,148-1,751,978 | 1398/158.74 | 5 | 4 |
| Py Alpha | PY17X_0106900 | coatomer alpha subunit, putative | *Plasmodium yoelii yoelii* | 01/264,465- 268,973 | 1380/ 160.18 | 4 | 3 |
| Pc Alpha | PCHAS_0106000 | coatomer alpha subunit, putative | *Plasmodium chabaudi chabaudi* | 01/ 229953 - 234466 | 1386/ 160.34 | 4 | 3 |
| Pb Alpha | PBANKA_0105400 | coatomer alpha subunit, putative | *Plasmodium berghei* | 1/203,081-207,619 | 1383/ 160.58 | 4 | 3 |
| Tg Alpha | TGGT1_240650 | putative coatomer protein complex, subunit alpha | *Toxoplasma gondii* | VI/1,326,973-1,343,296 | 1300/142.95 | 20 | 19 |
| Cp Alpha | cgd8_860 | coatomer protein complex subunit alpha, putative | *Cryptosporidium parvum* | 8/236,497-240,645 | 1382/ 156.34 | 1 | 0 |
| Bb Alpha | BBOV_III009950 | WD domain, G-beta repeat domain containing protein | *Babesia bovis* | 3/ 2,133,139-2,136,976 | 1266/143.80 | 2 | 1 |
| Ta Alpha | TA­11660 | coatomer alpha subunit, putative | *Theileria annulata* | 02/1651257-1655437 | 1279/147.71 | 7 | 6 |
| Nc Alpha | NCLIV_016850 | AT3G15980 protein, related | *Neospora caninum* | VI/1159952-1171542 | 1239/136.42 | 17 | 16 |
| Et Alpha | ETH_00021415 | coatomer alpha subunit, putative | *Eimeria tenella* | Scaff 21/ 60293-69363 | 1206/132.53 | 20 | 19 |
| Tp Alpha | TP02_0162 | coatomer alpha subunit, putative | *Theileria parva* | 2/ 317,561- 322,406 | 1358 /155.90 | 1 | 0 |

**Table S2:** The coatomer Beta homologues of apicomplexan parasite.

| Protein | Gene ID | Gene Name | Organisms | Location  (chr.no./position) | Size(protein)  aa/KDa | Exon (no.) | Intron (no.) |
| --- | --- | --- | --- | --- | --- | --- | --- |
| Pf Beta | PF3D7_1429800 | coatamer beta subunit, putative | *Plasmodium falciparum* | 14/ 1,170,829-1,175,293 | 1370/159.97 | 3 | 2 |
| Pc Beta | PCHAS_1015700 | coatamer protein, beta subunit, putative | *Plasmodium chabaudi chabaudi* | 10/ 638694 - 642630 | 1229/ 141.64 | 3 | 2 |
| Pb Beta | PBANKA_1014900 | coatamer protein, beta subunit, putative | *Plasmodium berghei* | 10/ 654,009 to 657,996 | 1256/ 145.62 | 1 | 0 |
| Pk Beta | PKNH_1328900 | coatamer protein, beta subunit, putative | *Plasmodium knowlesi* | 13/ 1329415 - 1333671 | 1325/ 149.13557 | 3 | 2 |
| Pv Beta | PVX_085050 | coatamer protein, beta subunit, putative | *Plasmodium vivax* | 13/ 870,150-74,849 | 1311/ 146.49 | 3 | 2 |
| Py Beta | PY17X_1016400 | coatamer protein, beta subunit, putative | *Plasmodium yoelii yoelii* | 10/660,686- 64,977 | 1286/ 148.48 | 3 | 2 |
| Tg Beta | TGGT1_266990 | beta-COP | *Toxoplasma gondii* | IX/ 687,782-98,362 | 1103/121.98 | 19 | 18 |
| Cp Beta | cgd6_273 | Coatomer_beta_subunit | *Cryptosporidium parvum* | 6/ 68,282-70,221 | 645/74.23 | 1 | 0 |
|  | cgd6_0263 | Coatomer_beta | *Cryptosporidium parvum* | 6/ 65,859-68,190 | 651/70.21 | 1 | 0 |
| Bb Beta | BBOV_IV010410 | coatamer beta subunit, putative | *Babesia bovis* | 1,422,438- ,425,522 | 993/110.89 | 4 | 3 |
| Ta Beta | TA02765 | coatamer, beta subunit, putative | *Theileria* *annulata* | 1/ 1179883-185333 | 992/111.48 | 7 | 6 |
| Nc Beta | NCLIV_039080 | Adaptin N terminal region family protein, related | *Neospora caninum* | IX/ 572225-581678 | 1117/123.26 | 19 | 18 |
| Tp Beta | TP01_0687 | Coatomer Beta subunt | *Theileria parva* | 1/1,444,309 to 1,448,884 | 923/105.86 | 10 | 9 |
| Et Beta | ETH_00026095 | coatomer beta subunit, putative | *Eimeria tenella* | scaff449/12-7351 | 753/79.86 | 13 | 12 |

**Table S3:** The coatomer Beta’ homologues of apicomplexan parasites.

| Protein | Gene ID | Gene name | Organism | Location(chr.no./position) | Size(protein) aa/KDa | Exon (no.) | Intron (no) |
| --- | --- | --- | --- | --- | --- | --- | --- |
| Pf Beta’ | PF3D7_0905900 | coatomer subunit beta, putative | *Plasmodium falciparum* | 9/ 293,585 to 296,617 | 1010/118.25 | 1 | 0 |
| Pc Beta’ | PCHAS_0416400 | coatomer subunit beta, putative | *Plasmodium chabaudi chabaudi* | 04/ 584,651 to 587,599 | 982/113.88 | 1 | 0 |
| Pb Beta’ | PBANKA_0415500 | coatomer subunit beta, putative | *Plasmodium berghei* | 04/ 552,926 to 555,925 | 999/116.60 | 1 | 0 |
| Pk Beta’ | PKNH_0703600 | beta subunit of coatomer complex, putative | *Plasmodium knowlesi* | 07/ 210,205 to 213,231 | 1008/116.41 | 1 | 0 |
| Pv Beta’ | PVX_098735 | coatomer complex beta subunit, putative | *Plasmodium vivax* | 07/ 199,603 to 202,644 | 1013/116.13 | 1 | 0 |
| Py Beta’ | PY17X_0418300 | coatomer subunit beta, putative | *Plasmodium yoelii yoelii* | 04/657,212 to 660,211 | 999/116.38 | 1 | 0 |
| Cp beta’ | cgd7_5010 | coatomer complex beta | *Cryptosporidium parvum* | 07/1,155,257 to 1,158,730 | 1157/131.94 | 1 | 0 |
| Tg Beta’ | TGGT1_235020 | putative COPI protein | *Toxoplasma gondii* | X/4,501,792 to 4,518,157 | 1256/137.08 | 22 | 21 |
| Bb Beta’ | BBOV_IV005890 | coatomer beta subunit, putative | *Babesia bovis* | Not Assigned/461,510 to 464,180 | 873/98.32 | 1 | 1 |
| Ta Beta’ | TA20925 | beta subunit of coatomer complex, putative | *Theileria annulata* | chr01.contig1/1805252-1808387 | 940/106.69 | 6 | 5 |
| Et Beta’ | ETH_00041840 | coatomer protein complex subunit beta, putative | *Eimeria tenella* | scaff3923/172-2928 | 376/40.05 | 8 | 7 |
|  | ETH_00016825 | hypothetical protein | *Eimeria tenella* | HG673814:40,043-41,375 | 212/23.23 | 3 | 2 |
| Nc Beta’ | NCLIV_049710 | Hypothetical protein | *Neospora caninum* | chrX/ 4210551-4222994 | 1299/ 140.89 | 22 | 21 |
| Tp Beta’ | TP01_0394 | coatomer beta subunit, putative | *Theileria parva* | 1/801,477 to 804,522 | 911/103.34 | 5 | 4 |

**Table S4:** The coatomer Gamma homologues of apicomplexan parasites.

| Protein | Gene ID | Gene Name | Organisms | Location  (chr.no./position) | Size(protein)  aa/KDa | Exon (no.) | Intron (no.) |
| --- | --- | --- | --- | --- | --- | --- | --- |
| Pf Gamma | PF3D7_1145100 | coatomer subunit gamma, putative | *Plasmodium falciparum* | 11/1,783,357-1,786,563 | 1068/124.37 | 1 | 0 |
| Pc Gamma | PCHAS_0705100 | coatomer subunit gamma, putative | *Plasmodium chabaudi chabaudi* | 7/ 211638 - 214646 | 1002/ 115.41 | 1 | 0 |
| Pb Gamma | PBANKA_0903900 | coatomer subunit gamma, putative | *Plasmodium berghei* | 9/174,480-177,419 | 979/113.49 | 1 | 0 |
| Pk Gamma | PKNH_0943000 | coatomer gamma subunit, putative | *Plasmodium knowlesi* | 9/ 1938298 - 1941297 | 999/ 113.97588 | 1 | 0 |
| Pv Gamma | PVX_092860 | coatomer subunit gamma, putative | *Plasmodium vivax* | 9/1,767,103-1,770,135 | 1010/ 114.53 | 1 | 0 |
| Py Gamma | PY17X_0905300 | coatomer subunit gamma, putative | *Plasmodium yoelii yoelii* | 9/220,598-223,624 | 1008/115.86 | 1 | 0 |
| Tg Gamma | TGGT1_273370 | putative coatomer gamma 2-subunit protein | *Toxoplasma gondii* | VIII/ 3,554,530- 3,558,659 | 1044/112.28 | 2 | 1 |
| Cp Gamma | cgd7_1910 | Coatomer_gamma_subunit/_adaptor_appendage/Ig-like_subdomain_containng_protein | *Cryptosporidium parvum* | 7/480,221- 483,031 | 936/104.80 | 1 | 0 |
| Bb Gamma | BBOV_II002960 | adaptin N terminal region family protein | *Babesia bovis* | 2/690,094-692,865 | 923/103.34 | 1 | 0 |
| Ta Gamma | TA07695 | coatamer, gamma subunit, putative | *Theileria annulata* | 04/594309-597080 | 923/104.68 | 1 | 0 |
| Et Gamma | ETH_00041395 | coatomer gamma 2-subunit protein, putative | *Eimeria tenella* | scaff3712/350-1080 | 178/17.89 | 2 | 1 |
| Nc Gamma | NCLIV_034270 | coatomer gamma 2-subunit protein, putative | *Neospora caninum* | VIII/3360706-3364262 | 1032/110.57 | 2 | 1 |
| Tp Gamma | TP04_0297 | coatomer gamma subunit, putative | *Theileria parva* | Not Assigned/584,436 to 587,219 | 927/105.00 | 1 | 0 |

**Table S5:** The coatomer Delta homologues of apicomlexan parasites.

| Protein | Gene ID | Gene Name | Organisms | Location  (chr.no./position) | Size(protein)  aa/KDa | Exon (no.) | Intron (no.) |
| --- | --- | --- | --- | --- | --- | --- | --- |
| Pf Delta | PF3D7_1134800 | coatomer subunit delta | *Plasmodium falciparum* | 11/ 1,367,186-1,368,809 | 487/56.84 | 2 | 1 |
| Pk Delta | PKNH_0933000 | coatomer subunit delta, putative | *Plasmodium knowlesi* | 9/1465466 – 1467019 | 517/59.82926 | 2 | 0 |
| Pv Delta | PVX_092350 | coatomer delta subunit, putative | *Plasmodium vivax* | 9/1,303,942-1,305,498 | 518/59.19 | 1 | 0 |
| Py Delta | PY17X_0915200 | coatomer subunit delta, putative | *Plasmodium yoelii yoelii* | 9/709340-710824 | 494/57.42 | 1 | 0 |
| Pc Delta | PCHAS_0932300 | coatomer subunit delta, putative | *Plasmodium chabaudi chabaudi* | 09/ 1176825 - 1178309 | 494/ 57.40 | 1 | 0 |
| Pb Delta | PBANKA_0913700 | coatomer subunit delta, putative | *Plasmodium berghei* | 09/ 517253 - 518737 | 494/ 57.50 | 1 | 0 |
| Tg Delta | TGGT1_221522 | adaptor complexes medium subunit family protein | *Toxoplasma gondii* | 2/ 393,309- 399,736 | 571/61.16 | 10 | 9 |
| Cp Delta | Cgd7_2940 | Coatomer complex delta chain | *Cryptosporidium parvum* | 7/ 694862 - 696469 | 535/ 60.71 | 1 | 0 |
| Bb Delta | BBOV_II001990 | conserved hypothetical protein | *Babesia bovis* | 2/478,905-480,602 | 498/56.12 | 6 | 5 |
| Ta Delta | TA07885 | coatomer delta subunit, putative | *Theileria annulata* | 4/ 660975-663005 | 496/57.28 | 9 | 8 |
| Et Delta | ETH_00011140 | coatomer delta subunit, putative | *Eimeria tenella* | scaff5/ 458114-461520 | 551 /60.53 | 10 | 9 |
| Nc Delta | NCLIV_005050 | coatomer delta subunit, putative | *Neospora caninum* | 2/407193-412395 | 634 /68.35 | 8 | 7 |
| Tp delta | TP04_0332 | Hypothetical protein | *Theileria pava* | Not Assigned/ 644455 - 645059 | 159/17.91 | 2 | 1 |
|  | TP04_0333 | Hypothetical protein | *Theileria pava* | Not Assigned/ 645198 - 646060 | 254/ 29.56 | 4 | 3 |

**Table S6:** The coatomer Epsilon homologues of apicomplexan parasites.

| Protein | Gene ID | Gene Name | Organisms | Location  (chr.no./position) | Size(protein)  aa/KDa | Exon  (no.) | Intron  (no.) |
| --- | --- | --- | --- | --- | --- | --- | --- |
| Pf Epsilon | PF3D7_0808400 | coatomer subunit epsilon, putative | *Plasmodium falciparum* | 8/423,527-424,536 | 278/33.09 | 2 | 1 |
| Pc Epsilon | PCHAS_1222900 | coatomer subunit epsilon, putative | *Plasmodium chabaudi chabaudi* | 12/820,117- 821,060 | 283/33.52 | 2 | 1 |
| Pb Epsilon | PBANKA_1222300 | coatomer subunit epsilon, putative | *Plasmodium berghei* | 12/ 821,778 | 283/33.67 | 2 | 1 |
| Pk Epsilon | PKNH_0110300 | coatomer subunit epsilon, putative | *Plasmodium knowlesi* | 1/485311 – 486339 | 291/ 34.23 | 2 | 1 |
| Pv Epsilon | PVX_088115 | coatomer epsilon subunit, putative | *Plasmodium vivax* | 1/440,314-441,364 | 290/33.56 | 2 | 1 |
| Py Epsilon | PY17X_1225500 | coatomer subunit epsilon, putative | *Plasmodium yoelii yoelii* | 12/945,423-946,927 | 283/33.82 | 2 | 1 |
| Cp Epsilon | cgd4_4100 | coatomer epsilon subunit | *Cryptosporidium parvum* | 4/1,004,816-1,005,754 | 312/36.29 | 1 | 0 |
| Tg Epsilon | TGGT1_244390 | coatomer epsilon subunit protein | *Toxoplasma gondii* | VI/3,145,659- 3,150,988 | 309/33.93 | 9 | 8 |
| Et Epsilon | ETH_00011095 | coatomer epsilon subunit, putative | *Eimeria tenella* | scaff5/375709-378972 | 306 /32.83 | 9 | 8 |
| Nc Epsilon | NCLIV_018950 | putative coatomer epsilon subunit | *Neospora caninum* | VI/ 2951632-2955986 | 276/30.60 | 8 | 7 |

**Table S7:** The coatomer Zeta homologues of apicomplexan parasites.

| Protein | Gene ID | Gene Name | Organisms | Location  (chr.no./position) | Size(protein)  aa/KDa | Exon  (no.) | Intron  (no.) |
| --- | --- | --- | --- | --- | --- | --- | --- |
| Pf Zeta | PF3D7_0415400 | coatomer subunit zeta, putative | *Plasmodium falciparum* | 4/686,414- 687,040 | 208/23.79 | 1 | 0 |
| Pc Zeta | PCHAS_0726500 | coatomer subunit zeta, putative | *Plasmodium chabaudi chabaudi* | 7/ 944,245-944,901 | 218/24.88 | 1 | 0 |
| Pb Zeta | PBANKA_0717400 | coatomer subunit zeta, putative | *Plasmodium berghei* | 7/598,462- 599,121 | 219/ 25.25 | 1 | 0 |
| Pk Zeta | PKNH_0507000 | coatomer subunit zeta, putative | *Plasmodium knowlesi* | 5/ 274428 - 275054 | 208/ 23.47872 | 1 | 0 |
| Pv Zeta | PVX_089725 | coatomer subunit zeta, putative | *Plasmodium vivax* | 5/ 790,309-790,935 | 208/ 23.62 | 1 | 0 |
| Py Zeta | PY17X_0717600 | coatomer subunit zeta, putative | *Plasmodium yoelii yoelii* | 7/635,488- 636,129 | 213/24.52 | 1 | 0 |
| Cp Zeta | cgd7_4180 | hypothetical protein | *Cryptosporidium parvum* | 7/940,557- 941,145 | 176/19.32 | 2 | 1 |
| Tg Zeta | TGGT1_280550 | clathrin adaptor complex small chain subfamily protein | *Toxoplasma gondii* | VIIa/287,174-289,721 | 207/21.61 | 5 | 4 |
| Bb Zeta | BBOV_III000560 | nonclathrin coat protein zeta2-cop-related protein, putative | *Babesia bovis* | 3/132165-133127 | 191/21.21 | 5 | 4 |
| Ta Zeta | TA18095 | Clathrin adapter complex-related protein, putative | *Theileria annulata* | 03/1627610-1629312 | 370/ 43.64 | 11 | 10 |
| Et Zeta | ETH_00027025 | coatomer zeta-2 subunit, putative | *Eimeria tenella* | supercontig Eth_scaff109/ 83623-84369 | 144/16.98 | 2 | 1 |
| Nc Zeta | NCLIV_019600 | hypothetical protein | *Neospora caninum* | VIIa/274146-276724 | 214/22.11 | 5 | 4 |
| Tp Zeta | TP03_0756 | nonclathrin coat protein, putative | *Theileria parva* | Not Assigned/ 286394 - 287154 | 190/ 21825.23 | 4 | 3 |

**Table S8:** Coatomer sec13 homologues of apicomplexan parasites.

| Protein | Gene ID | Gene Name | Organisms | Location  (chr.no./position) | Size(protein)  aa/KDa | Exon  (no.) | Intron  (no.) |
| --- | --- | --- | --- | --- | --- | --- | --- |
| Pf sec13 | PF3D7_1230700 | protein transport protein SEC13, putative | *Plasmodium falciparum* | 12/1,259,620-1,262,088 | 822/90.75 | 1 | 0 |
| Pc sec13 | PCHAS_1447600 | protein transport protein SEC13, putative | *Plasmodium chabaudi chabaudi* | 14/1737169-1739426 | 722/80.29 | 2 | 1 |
| Pb sec13 | PBANKA_1445400 | protein transport protein SEC13, putative | *Plasmodium berghei* | 14/1739873-1742022 | 687/76.70 | 2 | 1 |
| Pk sec13 | PKNH_1450100 | protein transport protein SEC13, putative | *Plasmodium knowlesi* | 14/2166583-2168829 | 748/79.96 | 1 | 0 |
| Pv sec13 | PVX_124175 | protein transport protein SEC13, putative | *Plasmodium vivax* | 14/ 2,108,558-2,110,849 | 763/81.08 | 1 | 0 |
| Py sec13 | PY17X_1447900 | protein transport protein SEC13, putative | *Plasmodium yoelii yoelii* | 14/1875827-1877993 | 690/ 77.38 | 2 | 1 |
| Tg sec13 | TGGT1_201700 | WD domain, G-beta repeat-containing protein | *Toxoplasma gondii* | VIIa/3,740,764-3,744,193 | 654/67.41 | 5 | 4 |
| Cp sec13 | cgd8_4110 | hypothetical protein | *Cryptosporidium parvum* | 8/1,029,493- 1,031,298 | 601/63.72 | 1 | 0 |
| Bb sec13 | Absent |  | *Babesia bovis* |  |  |  |  |
| Ta sec13 | Absent |  | *Theileria annulata* |  |  |  |  |
| Et sec13 | ETH_00020090 | sec 13, putative | *Eimeria tenella* | supercontig Eth_scaff106/18821-20341 | 385/40.87 | 1 | 0 |
| Nc sec13 | NCLIV_023040 | Hypothetical protein | *Neospora caninum* | VIIa/3,326,636..3,329,857 | 645/66.24 | 4 | 3 |
| Tp | Absent |  | *Theileria parva* |  |  |  |  |

**Table S9:** The Coatomer sec31 Homologues of apicomplexan parasites.

| Protein | Gene ID | Gene name | Organisms | Location  (chr.no./position) | Size(protein)  aa/KDa | Exon  (no.) | Intron  (no.) |
| --- | --- | --- | --- | --- | --- | --- | --- |
| Pf sec31 | PF3D7_0214100 | protein transport protein SEC31 | *Plasmodium falciparum* | 2/ 568,529 to 573,315 | 1471/166.71 | 4 | 3 |
| Pc sec31 | PCHAS_031310 | protein transport protein SEC31, putative | *Plasmodium chabaudi chabaudi* | 3/447,870 to 452,460 | 1394/ 155.87 | 4 | 3 |
| Pb sec31 | PBANKA_0311000 | protein transport protein SEC31, putative | *Plasmodium berghei* | 3/ 381,422 to 385,971 | 1388/156.64 | 4 | 3 |
| Pk sec31 | PKNH_0406700 | protein transport protein Sec31, putative | *Plasmodium knowlesi* | 4/266225-270958 | 1416/156.25 | 4 | 3 |
| Pv sec31 | PVX_002830 | protein transport protein SEC31, putative | *Plasmodium vivax* | 4/307,397 to 312,265 | 1438/ 154.85 | 4 | 3 |
| Py sec31 | PY17X_0311500 | protein transport protein SEC31, putative | *Plasmodium yoelii yoelii* | 3/411,411 to 415,980 | 1393/157.18 | 4 | 3 |
| Tg sec31 | TGGT1_311400 | WD domain, G-beta repeat-containing protein | *Toxoplasma gondii* | XI/ 2010353 - 2023624 | 1585/ 165.58 | 16 | 15 |
| Cp sec31 | cgd4_260 | WD repeat protein | *Cryptosporidium parvum* | 4/ 68336 - 72415 | 1359/ 148.68 | 1 | 0 |
| Bb sec31 | BBOV_IV008580 | Conserved hypothetical protein | *Babesia bovis* | Not Assigned/ 1038037 – 1041143 | 1002/ 109.19 | 1 | 0 |
| Ta sec31 | TA06395 | Hypothetical protein, conserved | *Theileria annulata* | 1/ 1942712 - 1946688 | 1296/ 140.93 | 2 | 1 |
| Et sec31 | ETH_00026970 | Hypothetical protein, conserved | *Eimeria tenella* | Not Assigned/213452 - 221762 | 1431/ 152.95 | 18 | 17 |
| Nc sec31 | NCLIV_055300 | Hypothetical protein | *Neospora caninum* | XI/ 1812763 - 1824017 | 1484/ 155.07 | 15 | 14 |
| Tp sec31 | TP01_0918 | Hypothetical protein | *Theileria parva* | 1/ 1896840 - 1900874 | 1310/ 141.76 | 2 | 1 |

**Table S10:** The coatomer sec23 homologues of apicomplexan parasites.

| Protein | Gene ID | Gene Name | Organisms | Location  (chr.no./position) | Size(protein)  aa/KDa | Exon  (no.) | Intron  (no.) |
| --- | --- | --- | --- | --- | --- | --- | --- |
| Pf sec23 | PF3D7_0822600 | protein transport protein SEC23 | *Plasmodium falciparum* | 08/ 997055 - 999529 | 759/ 86.17 | 2 | 1 |
| Pc sec23 | PCHAS_0717300 | protein transport protein SEC23, putative | *Plasmodium chabaudi chabaudi* | 07/ 645966 - 648473 | 759/ 86.25 | 2 | 1 |
| Pb sec23 | PBANKA_0708000 | protein transport protein SEC23, putative | *Plasmodium berghei* | 07/ 330576 - 332985 | 759/ 86.14 | 2 | 1 |
| Pk sec23 | PKNH_1316600 | protein transport protein SEC23, putative | *Plasmodium knowlesi* | 13/ 750662 - 753125 | 759/ 86.20 | 2 | 1 |
| Pv sec23 | PVX_089235 | protein transport protein SEC23, putative | *Plasmodium vivax* | 05/ 403416 - 405866 | 759/ 86.22 | 2 | 1 |
| Py sec23 | \| PY17X_0708300 \|  \| \| --- \| --- \| | protein transport protein SEC23, putative | *Plasmodium yoelii yoelii* | 07/ 322116 - 324515 | 759/ 86.24 | 2 | 1 |
| Tg sec23 | TGGT1_291680 | Sec23/Sec24 trunk domain-containing protein | *Toxoplasma gondii* | IX/ 4131104 - 4145209 | 791/ 87.60 | 15 | 14 |
| Cp sec23 | Cgd3_1820 | putative Sec23 | *Cryptosporidium parvum* | 3/ 487048 – 489324 | 758/ 84.85 | 1 | 0 |
| Bb sec23 | BBOV_II007590 | sec23 protein | *Babesia bovis* | 2/1682162-1684669 | 770/86.13 | 2 | 1 |
| Ta sec23 | TA14650 | protein transport protein (SEC23 homologue), putative | *Theileria annulata* | 2/594494-596934 | 774/86.57 | 2 | 1 |
| Et sec23 | ETH_00006120 | protein transport protein Sec23, putative | *Eimeria tenella* | Not Assigned/ 142743 - 153812 | 874/ 96.72 | 18 | 17 |
| Nc sec23 | NCLIV_043180 | hypothetical protein | *Neospora caninum* | IX/ 3950453 - 3959154 | 795/ 88.00 | 15 | 14 |
| TP sec13 | TP02_0701 | Sec23, putative | *Theileria parva* | 2/1398233-1400989 | 774/86.32 | 2 | 1 |

**Table S11:** The coatomer sec24A homologues of apicomplexan parasites.

| Protein | Gene ID | Gene Name | Organisms | Location  (chr.no./position) | Size(protein)  aa/KDa | Exon  (no.) | Intron  (no.) |
| --- | --- | --- | --- | --- | --- | --- | --- |
| Pb SEC24A | PBANKA_1137200 | protein transport protein Sec24A, putative | *Plasmodium berghei* | 11/ 1405856 - 1409543 | 942/ 106.43 | 5 | 4 |
| Pc SEC24A | PCHAS_1136700 | protein transport protein Sec24A, putative | *Plasmodium chabaudi* | 11/ 1333916 - 1337573 | 941/ 106.03 | 5 | 4 |
| Pf SEC24A | PF3D7_1361100 | protein transport protein Sec24A | *Plasmodium falciparum* | 13/ 2450461 - 2453760 | 940/ 106.68 | 5 | 4 |
| Pk SEC24A | PKNH_1110300 | protein transport protein Sec24A, putative | *Plasmodium knowlesi* | 11/ 431927 - 435605 | 933/ 104.28 | 5 | 4 |
| Pv SEC24A | PVX_115015 | protein transport protein Sec24A, putative | *Plasmodium vivax* | 11/ 392868 - 396525 | 935/ 104.31 | 5 | 4 |
| Py SEC24A | PY17X_1138600 | protein transport protein Sec24A, putative | *Plasmodium yoelii* | 11/ 1588641-1592317 | 942/ 106.25 | 5 | 4 |
| Tg sec24A | TGGT1_277000 | putative transport protein Sec24 | *Toxoplasma gondii* | XII/ 6,701,488 to 6,710,271 | 1019/108.03 | 9 | 8 |
| Cp sec24A | cgd8_4470 | hypothetical protein | *Cryptosporidium parvum* | 8/1,102,950 to 1,105,636 | 874/97.38 | 2 | 1 |
| Bb sec24A | BBOV_IV000740 | Sec 24 protein transport protein, putative | *Babesia bovis* | 175,794 to 178,516 | 835/ 91.33 | 1 | 0 |
| Ta sec24A | TA20050 | protein transport protein sec24-like, putative | *Theileria annulata* | chr01.contig1/2376580-2379863 | 905/100.34 | 11 | 10 |
| Et sec24A | ETH_00007535 | transport protein Sec24, putative | *Eimeria tenella* | Eth_scaff381/474-6220 | 706/73.41 | 7 | 6 |
| Nc sec24A | NCLIV_068580 | hypothetical protein | *Neospora caninum* | XII/ 6387494 - 6393091 | 603/ 64.85 | 10 | 9 |
| Tp sec24A | TP01_0099 | vesicle transport protein, putative | *Theileria parva* | 1/ 201664 - 205011 | 899/ 99.72 | 11 | 10 |

**Table S12:** The coatomer sec24B homologues of apicomplexan parasites.

| Protein | Gene ID | Gene Name | Organism | Location  (chr.no./position) | Size(protein) aa/KDa | Exon (no.) | Intron (no.) |
| --- | --- | --- | --- | --- | --- | --- | --- |
| Pb Sec24B | PBANKA_1002800 | protein transport protein Sec24B, putative | *Plasmodium berghei* | 10/ 171451 - 176105 | 1381/ 158.42 | 5 | 4 |
| Pc Sec24B | PCHAS_1003700 | protein transport protein Sec24B, putative | *Plasmodium chabaudi chabaudi* | 10/ 164409 - 169072 | 1381/ 157.41 | 5 | 4 |
| Pf Sec24B | PF3D7_0405100 | protein transport protein Sec24B | *Plasmodium falciparum* | 04/ 272255 - 276824 | 1350/ 155.94 | 5 | 4 |
| Pk Sec24B | PKNH_0303200 | protein transport protein Sec24B, putative | *Plasmodium knowlesi* | 03/ 161971 - 167246 | 1532/ 170.91 | 5 | 4 |
| Pv Sec24B | PVX_000985 | protein transport protein Sec24B, putative | *Plasmodium vivax* | 03/ 133637 - 139052 | 1554/ 170.64 | 5 | 4 |
| Py Sec24B | PY17X_1004200 | protein transport protein Sec24B, putative | *Plasmodium yoelii* | 10/ 279869-284506 | 1372/ 157.13 | 5 | 4 |
| Tg sec24B | TGGT1_226510 | Sec23/Sec24 trunk domain-containing protein | *Toxoplasma gondii* | X/ 1468057 - 1478151 | 1540/ 165.25 | 14 | 13 |
| Cp sec24B | cgd8_1250 | SEC24C-like component of COPII coatamer of ER-golgi vesicles | *Cryptosporidium parvum* | 8/ 338832 - 342284 | 1150/ 127.84 | 1 | 0 |
| Et sec24B | ETH_00026990 | sec23/Sec24 helical domain-containing protein, putative | *Eimeria tenella* | Not Assigned/ 246448 - 254625 | 1483/ 165.35 | 17 | 16 |
| Nc sec24B | NCLIV_046450 | putative sec23/Sec24 helical domain-containing protein | *Neospora caninum* | x/ 1386494 - 1395654 | 1517/ 162.51 | 14 | 13 |
| Ta sec24B | TA21015 | protein transport protein sec24-like, putative | *Theileria annulata* | 1/ 760501 - 763736 | 1051/ 118.81 | 2 | 1 |
| Bb sec24B | BBOV_IV005730 | conserved hypothetical protein | *Babesia bovis* | Not Assigned/ 429141 - 432475 | 1024/ 115.35 | 1 | 0 |
| Tp sec24B | TP01_0373 | hypothetical protein | *Theileria parva* | 1/ 759455 - 762706 | 1034/ 116.76 | 2 | 1 |

**Table S13:** The Coatomer Sar1a homologues of apicomplexan parasites.

| Protein | Gene ID | Gene Name | Organism | Location  (chr.no./position) | Size(protein)  aa/KDa | Exon  (no.) | Intron  (no.) |
| --- | --- | --- | --- | --- | --- | --- | --- |
| Pf sar1a | PF3D7_0416800 | small GTP-binding protein sar1 | *Plasmodium falciparum* | 4/729,962 to 731,223 | 192/22.02 | 3 | 2 |
| Pc sar1a | PCHAS_0727900 | small GTP-binding protein sar1, putative | *Plasmodium chabaudi chabaudi* | 7/979,597 to 980,593 | 191/21.90 | 3 | 2 |
| Pb sar1a | PBANKA_0718800 | small GTP-binding protein sar1, putative | *Plasmodium berghei* | 7/635,357 to 636,357 | 191/21.92 | 3 | 2 |
| Pk sar1a | PKNH_0509200 | small GTP-binding protein sar1, putative | *Plasmodium knowlesi* | 5/ 386418 - 387564 | 191/ 21.94 | 3 | 2 |
| Pv sar1a | PVX_089930 | small GTP-binding protein sar1, putative | *Plasmodium vivax* | 5/946,227 to 947,218 | 191/21.94 | 3 | 2 |
| Py sar1a | PY17X_0718900 | small GTP-binding protein sar1, putative | *Plasmodium yoelii yoelii* | 7/845224-846284 | 191/ 21.91 | 3 | 2 |
| Tg sar1a | TGGT1_215060 | putative small GTP-binding protein sar1 | *Toxoplasma gondii* | X/6,612,856 to 6,615,947 | 192/22.14 | 7 | 6 |
| Cp sar1a | cgd7_2330 | SAR1-like small GTpase | *Cryptosporidium parvum* | 7/ 555,755 to 556,390 | 211/24.13 | 1 | 0 |
| Bb sar1a | BBOV_II004300 | small GTP-binding protein sar1 | *Babesia bovis* | 2/1,006,991 to 1,008,048 | 197/22.23 | 3 | 2 |
| Ta sar1a | TA08955 | small GTPase, putative | *Theileria annulata* | 4/ 1090292 - 1090945 | 195/22.06 | 2 | 1 |
| Et sar1a | ETH_00027560 | small GTP-binding protein sar1, putative | *Eimeria tenella* | Not Assigned/ 1834 - 3028 | 116/13.44 | 4 | 3 |
| Nc sar1a | NCLIV_052070 | hypothetical protein | *Neospora caninum* | X/ 6251955-6254550 | 192/22.14 | 7 | 6 |
| Tp sar1a | TP04_0542 | GTP-binding protein, putative | *Theileria parva* | Not Assigned/ 1080159 - 1080821 | 195/22.06 | 2 | 1 |

**Table S14:** The domain architecture of the COPI and COPII subunits of apicomplexan parasites in comparison to that of the human homologs. The number of domain(s) are mentioned for the corresponding proteins.

| **COPI** | | | | | | | | | | |
| --- | --- | --- | --- | --- | --- | --- | --- | --- | --- | --- |
| **Alpha(Human)**  **WD40**  **ANAPC4_WD40**  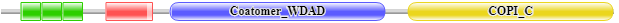 | | | | | | | | | | |
| **Organism** | **WD40** | | | **ANAPC4_WD40** | | | | **Coatomer_WDAD** | **COPI_C** | |
| ***Homo sapience*** | **3 (41-79, 83-121, 125-163)** | | | **1 (211-303)** | | | | **1 (338-767)** | **1 (815-1224)** | |
| ***Arabidopsis thaliana*** | **3 (41-79, 83-121, 125-163)** | | | **1 (210-303)** | | | | **1 (337-769)** | **1 (815-1216)** | |
| ***Saccharomyces cerevisiae*** | **5 (43-81, 85-123, 127-165, 197-237, 243-281)** | | | **-** | | | | **1 (343-771)** | **1 (816-1201)** | |
| ***Plasmodium falciparum*** | **5 (41-79, 83-121, 125-163, 260-298, 304-343)** | | | **-** | | | | **1 (591-990)** | **1 (1106-1511)** | |
| ***Plasmodium knowlesi*** | **5 (41-79, 83-121, 125-163, 236-273, 279-318)** | | | **-** | | | | **1 (498-890)** | **1 (981-1364)** | |
| ***Plasmodium vivax*** | **5 (41-79, 83-121, 125-163, 239-276, 282-321)** | | | **-** | | | | **1 (535-923)** | **1 (1021-1397)** | |
| ***Plasmodium yoelii yoelii*** | **5 (41-79, 83-121, 125-163, 238-276, 282-321)** | | | **-** | | | | **1 (482-886)** | **1 (961-1379)** | |
| ***Plasmodium chabaudi chabaudi*** | **5 (41-79, 83-121, 125-163, 239-276, 282-321)** | | | **-** | | | | **1 (493-888)** | **1 (992-1385)** | |
| ***Plasmodium berghei*** | **5 (41-79, 83-121, 125-163, 238-275, 281-320)** | | | **-** | | | | **1 (493-888)** | **1 (958-1382)** | |
| ***Toxoplasma gondii*** | **6 (5-40, 44-82, 86-124, 128-166, 206-244, 250-288)** | | | **-** | | | | **1 (444-823)** | **1 (916-1300)** | |
| ***Cryptosporidium parvum*** | **5 (41-79, 83-121, 125-163, 204-241, 247-285)** | | |  | | | | **1 (573-890)** | **1 (1062-1382)** | |
| ***Babesia bovis*** | **5 (41-79, 83-121, 125-163, 218-255, 261-300)** | | | **-** | | | | **1 (448-886)** | **1 (993-1265)** | |
| ***Theileria annulata*** | **5 (41-79, 83-121, 125-163, 207-243, 249-285)** | | | **-** | | | | **1 (478-768)** | **1 (1020-1278)** | |
| ***Eimeria tenella*** | **3 (63-110, 135-173, 179-217)** | | | **-** | | | | **1 (383-735)** | **1 (807-1206)** | |
| ***Neospora caninum*** | **4 (5-40, 44-82, 86-124, 128-166, 206-244)** | | | **1 (223-329)** | | | | **1 (451-817)** | **1 (870-1199)** | |
| ***Theileria parva*** | **5 (41-79, 83-121, 125-163, 207-243, 249-285)** | | | **-** | | | | **1 (554-911)** | **1 (1102-1357)** | |
| ***Phytophthora sojae*** | **5 (41-79, 83-121, 125-163, 203-239, 245-283)** | | | **-** | | | | **1 (344-783)** | **1 (837-1246) (additional STIL_N domain (1508-1771))** | |
| **Beta (Human)**  **Coatomer beta C**  **Coatomer b Cpla**  **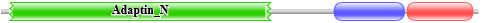** | | | | | | | | | | |
| **Organism** | **Adaptin N** | | | | | | | **Coatomer beta C** | **Coatomer b Cpla** | |
| ***Homo sapience*** | **1 (18-533)** | | | | | | | **1 (667-807)** | **1 (813-944)** | |
| ***Arabidopsis thaliana*** | **1 (15-522)** | | | | | | | **1 (669-808)** | **1 (813-940)** | |
| ***Saccharomyces cerevisiae*** | **1 (20-560)** | | | | | | | **1 (684-824)** | **1 (829-958)** | |
| ***Plasmodium falciparum*** | **1 (20-547)** | | | | | | | **1 (1029-1192)** | **1 (1197-1359)** | |
| ***Plasmodium knowlesi*** | **1 (20-519)** | | | | | | | **1 (978-1146)** | **1 (1151-1313)** | |
| ***Plasmodium vivax*** | **1 (20-492)** | | | | | | | **1 (949-1132)** | **1 (1137-1299)** | |
| ***Plasmodium yoelii yoelii*** | **1 (21-460)** | | | | | | | **1 (945-1107)** | **1 (1112-1274)** | |
| ***Plasmodium chabaudi chabaudi*** | **1 (22-654)** | | | | | | | **1 (888-1050)** | **1 (1055-1217)** | |
| ***Plasmodium berghei*** | **1 (20-459)** | | | | | | | **1 (915-1077)** | **1 (1082-1244)** | |
| ***Toxoplasma gondii*** | **1 (17-492)** | | | | | | | **1 (766-917)** | **1 (922-1094)** | |
| ***Cryptosporidium parvum*** |  | | | | | | | **1 (213-369)** | **2 (397-479, 481-582)** | |
|  | **2 (18-382, 464-591)** | | | | | | |  |  | |
| ***Babesia bovis*** | **1 (22-504)** | | | | | | | **1 (667-821)** | **1 (827-984)** | |
| ***Theileria annulata*** | **1 (1-348)** | | | | | | | **1 (674-788)** | **1 (794-887)** | |
| ***Eimeria tenella*** |  | | | | | | | **1 (414-559)** | **2 (571-672, 661-742)** | |
| ***Neospora caninum*** | **1 (17-492)** | | | | | | | **1 (789-930)** | **1 (935-1108)** | |
| ***Theileria parva*** | **1 (21-537)** | | | | | | | **1 (621-764)** | **1 (770-915)** | |
| ***Phytophthora sojae*** | **1 (24-517)** | | | | | | | **1 (705-846)** | **1 (851-978)** | |
| **Human(beta’)**    **WD40**  **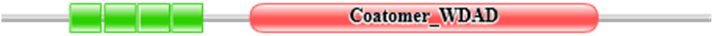** | | | | | | | | | | |
| **Organism** | **WD40** | | | | | | | **Coatomer_WDAD** | | |
| ***Homo sapience*** | **4 (89-127, 132-171, 175-215, 219-257)** | | | | | | | **1 (319-763)** | | |
| ***Arabidopsis thaliana*** | **4 (89-127, 132-171, 175-215, 219-257)** | | | | | | | **1 (319-764)** | | |
| ***Saccharomyces cerevisiae*** | **4 (87-125, 130-169, 174-214, 218-256)** | | | | | | | **1 (325-777)** | | |
| ***Plasmodium falciparum*** | **5 (47-85, 89-127, 132-170, 180-220, 224-262)** | | | | | | | **1 (323-806)** | | |
| ***Plasmodium knowlesi*** | **4 (89-127, 132-170, 180-220, 224-262)** | | | | | | | **1 (323-790)** | | |
| ***Plasmodium vivax*** | **4 (89-127, 132-170, 180-220, 224-262)** | | | | | | | **1 (323-791)** | | |
| ***Plasmodium yoelii yoelii*** | **4 (89-127, 132-170, 180-220, 224-262)** | | | | | | | **1 (323-785)** | | |
| ***Plasmodium chabaudi chabaudi*** | **4 (89-127, 132-170, 180-220, 224-262)** | | | | | | | **1 (323-782)** | | |
| ***Plasmodium berghei*** | **4 (89-127, 132-170, 180-220, 224-262)** | | | | | | | **1 (323-785)** | | |
| ***Toxoplasma gondii*** | **3 (89-128, 133-171, 192-231)** | | | | | | | **1 (367-825)** | | |
| ***Cryptosporidium parvum*** | **3 (93-131, 189-229, 233-271)** | | | | | | | **1 (337-836)** | | |
| ***Babesia bovis*** | **3 (89-127, 187-227, 231-269)** | | | | | | | **1 (331-791)** | | |
| ***Theileria annulata*** | **4 (89-127, 132-171, 221-261, 265-303)** | | | | | | | **1 (363-870)** | | |
| ***Eimeria tenella*** | **2 (25-65, 69-111)** | | | | | | | **1 (181-353)** | | |
|  | **2 (89-128, 133-171)** | | | | | | |  | | |
| ***Neospora caninum*** | **3 (87-128, 133-171, 197-236)** | | | | | | | **1 (372-828)** | | |
| ***Theileria parva*** | **4 (89-127, 132-171, 221-261, 265-303)** | | | | | | | **1 (363-884)** | | |
| ***Phytophthora sojae*** | **4 (89-127, 132-170, 176-215, 219-257)** | | | | | | | **1 (321-781)** | | |
| **Gamma(Human)**  **Cop-gamma platf Cpla**  **Coatomer g Cpla**  **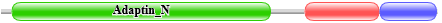** | | | | | | | | | | |
| **Organism** | **Adaptin N** | | | | | | **Cop-gamma platf** | | | **Coatomer g cpla** |
| ***Homo sapience*** | **1 (23-539)** | | | | | | **1 (609-756)** | | | **1 (758-870)** |
| ***Arabidopsis thaliana*** | **1 (25-543)** | | | | | | **1 (620-767)** | | | **1 (769-883)** |
| ***Saccharomyces cerevisiae*** | **1 (19-560)** | | | | | | **1 (669-819)** | | | **1 (821-933)** |
| ***Plasmodium falciparum*** | **1 (32-653)** | | | | | | **1 (781-949)** | | | **1 (951-1067)** |
| ***Plasmodium knowlesi*** | **1 (32-625)** | | | | | | **1 (727-880)** | | | **1 (882-998)** |
| ***Plasmodium vivax*** | **1 (32-630)** | | | | | | **1 (736-891)** | | | **1 (893-1009)** |
| ***Plasmodium yoelii yoelii*** | **1 (34-592)** | | | | | | **1 (704-889)** | | | **1 (891-1007)** |
| ***Plasmodium chabaudi chabaudi*** | **1 (33-596)** | | | | | | **1 (716-883)** | | | **1 (885-1001)** |
| ***Plasmodium berghei*** | **1 (31-596)** | | | | | | **1 (693-860)** | | | **1 (862-978)** |
| ***Toxoplasma gondii*** | **1 (37-594)** | | | | | | **1 (776-924)** | | | **1 (926-1043)** |
| ***Cryptosporidium parvum*** | **1 (26-558)** | | | | | | **1 (671-815)** | | | **1 (817-933)** |
| ***Babesia bovis*** | **1 (19-537)** | | | | | | **1 (663-799)** | | | **1 (801-922)** |
| ***Theileria annulata*** | **1 (19-552)** | | | | | | **1 (680-810)** | | | **1 (812-922)** |
| ***Eimeria tenella*** |  | | | | | |  | | | **1 (52-177)** |
| ***Neospra caninum*** | **1 (36-594)** | | | | | | **1 (768-913)** | | | **1 (915-1031)** |
| ***Theileria parva*** | **1 (19-549)** | | | | | | **1 (676-814)** | | | **1 (816-926)** |
| ***Phytophthora sojae*** | **1 (51-572)** | | | | | | **1 (644-793)** | | | **1 (795-909)** |
| **Delta(Human)**  **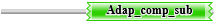** | | | | | | | | | | |
| **Organism** | **Adap comp sub** | | | | | | | | | |
| ***Homo sapience*** | **1 (174-422)** | | | | | | | | | |
| ***Arabidopsis thaliana*** | **1 (273-526)** | | | | | | | | | |
| ***Saccharomyces cerevisiae*** | **Clat_adaptor_s domain 1 (2-156)** | | | | | | | | | |
| ***Plasmodium falciparum*** | **-** | | | | | | | | | |
| ***Plasmodium knowlesi*** | **1 (281-515)** | | | | | | | | | |
| ***Plasmodium vivax*** | **1 (282-516)** | | | | | | | | | |
| ***Plasmodium yoelii yoelii*** | **-** | | | | | | | | | |
| ***Plasmodium chabaudi chabaudi*** | **-** | | | | | | | | | |
| ***Plasmodium berghei*** | **-** | | | | | | | | | |
| ***Toxoplasma gondii*** | **-** | | | | | | | | | |
| ***Cryptosporidium parvum*** | **-** | | | | | | | | | |
| ***Babesia bovis*** | **-** | | | | | | | | | |
| ***Theileria annulata*** | **1 (220-413)** | | | | | | | | | |
| ***Eimeria tenella*** | **1 (310-541)** | | | | | | | | | |
| ***Neospora caninum*** | **1 (335-558)** | | | | | | | | | |
| ***Theileria parva*** | **-** | | | | | | | | | |
| ***Phytophthora sojae*** | **1 (290-536)** | | | | | | | | | |
| **Epsilon(Human)**  **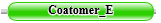** | | | | | | | | | | |
| **Organism** | **Coatomer E** | | | | | | | | | |
| ***Homo sapience*** | **1 (15-305)** | | | | | | | | | |
| ***Arabidopsis thaliana*** | **1 (7-293)** | | | | | | | | | |
| ***Saccharomyces cerevisiae*** | **1 (2-293)** | | | | | | | | | |
| ***Plasmodium falciparum*** | **1 (2-278)** | | | | | | | | | |
| ***Plasmodium knowlesi*** | **1 (2-291)** | | | | | | | | | |
| ***Plasmodium vivax*** | **1 (3-290)** | | | | | | | | | |
| ***Plasmodium yoelii yoelii*** | **1 (1-283)** | | | | | | | | | |
| ***Plasmodium chabaudi chabaudi*** | **1 (1-283)** | | | | | | | | | |
| ***Plasmodium berghei*** | **1 (1-283)** | | | | | | | | | |
| ***Toxoplasma gondii*** | **1 (2-297)** | | | | | | | | | |
| ***Cryptosporidium parvum*** | **1 (2-312)** | | | | | | | | | |
| ***Eimeria tenella*** | **1 (68-277)** | | | | | | | | | |
| ***Neospora caninum*** | **1 (2-184)** | | | | | | | | | |
| ***Phytophthora sojae*** | **1 (3-292)** | | | | | | | | | |
| **Zeta(Human)**  **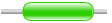**  **Clat adaptor S** | | | | | | | | | | |
| **Organism** | **Clat adaptor S** | | | | | | | | | |
| ***Homo sapience*** | **1 (45-185)** | | | | | | | | | |
| ***Arabidopsis thaliana*** | **1 (6-149)** | | | | | | | | | |
| ***Saccharomyces cerevisiae*** | **1 (8-159)** | | | | | | | | | |
| ***Plasmodium falciparum*** | **1 (8-182)** | | | | | | | | | |
| ***Plasmodium knowlesi*** | **1 (8-183)** | | | | | | | | | |
| ***Plasmodium vivax*** | **1 (8-183)** | | | | | | | | | |
| ***Plasmodium yoelii yoelii*** | **1 (47-188)** | | | | | | | | | |
| ***Plasmodium chabaudi chabaudi*** | **1 (47-192)** | | | | | | | | | |
| ***Plasmodium berghei*** | **1 (8-194)** | | | | | | | | | |
| ***Toxoplasma gondii*** | **1 (46-168)** | | | | | | | | | |
| ***Cryptosporidium parvum*** | **1 (5-154)** | | | | | | | | | |
| ***Babesia bovis*** | **1 (8-166)** | | | | | | | | | |
| ***Theileria annulata*** | **1 (8-168) with additional AP2 domain (266-324)** | | | | | | | | | |
| ***Eimeria tenella*** | **-** | | | | | | | | | |
| ***Neospora caninum*** | **1 (47-170)** | | | | | | | | | |
| ***Theileria parva*** | **-** | | | | | | | | | |
| ***Phytophthora sojae*** | **1 (8-153)** | | | | | | | | | |
|  |  | | | | | | | | | |
|  |  | | | | | | | | | |
| **COPII** | | | | | | | | | | |
| **Sec13(Human)**  **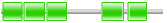**  **WD40** | | | | | | | | | | |
| **Organism** | **WD40** | | | | | | | | | |
| ***Homo sapience*** | **5 (4-41, 47-87, 93-131, 202-244, 254-290)** | | | | | | | | | |
| ***Arabidopsis thaliana*** | **5 (2-37, 46-86, 93-133, 140-192, 200-242)** | | | | | | | | | |
| ***Saccharomyces cerevisiae*** | **2 (43-83, 194-235)** | | | | | | | | | |
| ***Plasmodium falciparum*** | **2 (4-40, 47-87)** | | | | | | | | | |
| ***Plasmodium knowlesi*** | **2 (2-40, 47-87)** | | | | | | | | | |
| ***Plasmodium vivax*** | **2 (2-40, 47-87)** | | | | | | | | | |
| ***Plasmodium yoelii yoelii*** | **3 (2-40, 47-87, 145-201)** | | | | | | | | | |
| ***Plasmodium chabaudi chabaudi*** | **3 (3-40, 48-87, 145-201)** | | | | | | | | | |
| ***Plasmodium berghei*** | **3 (2-40, 47-87, 145-201)** | | | | | | | | | |
| ***Toxoplasma gondii*** | **4 (5-43, 61-101, 177-224, 233-283)** | | | | | | | | | |
| ***Cryptosporidium parvum*** | **4 (2-41, 48-87, 140-193, 203-245)** | | | | | | | | | |
| ***Eimeria tenella*** | **4 (3-41, 49-89, 98-137, 147-192)** | | | | | | | | | |
| ***Neospora caninum*** | **4 (4-43, 65-105, 187-232, 240-290)** | | | | | | | | | |
| ***Phytophthora sojae*** | **1 (230-273)** | | | | | | | | | |
| **Sec31(Human)**  **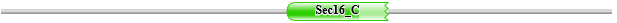** | | | | | | | | | | |
| **Organism** | **Sec16 C** | | | | | | | | | |
| ***Homo sapience*** | **1 (573-771)** | | | | | | | | | |
| ***Arabidopsis thaliana*** | **1 WD40 (105-144) 1 Sec16C (503-702)** | | | | | | | | | |
| ***Saccharomyces cerevisiae*** | **2 Wd40 (102-137, 248-286), 1 Sec16C (514-679), 1 Sec31 (899-946), 1 SRA1 (1141-1266)** | | | | | | | | | |
| ***Plasmodium falciparum*** | **1 (734-942)** | | | | | | | | | |
| ***Plasmodium knowlesi*** | **1 (722-928)** | | | | | | | | | |
| ***Plasmodium vivax*** | **-** | | | | | | | | | |
| ***Plasmodium yoelii yoelii*** | **1 (704-909)** | | | | | | | | | |
| ***Plasmodium chabaudi chabaudi*** | **1 (709-922)** | | | | | | | | | |
| ***Plasmodium berghei*** | **1 (703-915)** | | | | | | | | | |
| ***Toxoplasma gondii*** | **-** | | | | | | | | | |
| ***Cryptosporidium parvum*** | **-** | | | | | | | | | |
| ***Babesia bovis*** | **-** | | | | | | | | | |
| ***Theileria annulata*** | **-** | | | | | | | | | |
| ***Eimeria tenella*** | **-** | | | | | | | | | |
| ***Neospora caninum*** | **-** | | | | | | | | | |
| ***Theileria parva*** | **-** | | | | | | | | | |
| ***Phytophthora sojae*** | **1 WD40 (173-211) 1 Sec16C (601-798)** | | | | | | | | | |
| **Sar1a (Human)**  **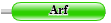** | | | | | | | | | | |
| **Organism** | **Arf** | | | | | | | | | |
| ***Homo sapience*** | **1 (12-197)** | | | | | | | | | |
| ***Arabidopsis thaliana*** | **1 (7-192)** | | | | | | | | | |
| ***Saccharomyces cerevisiae*** | **1 (10-190)** | | | | | | | | | |
| ***Plasmodium falciparum*** | **1 (7-191)** | | | | | | | | | |
| ***Plasmodium knowlesi*** | **1 (7-190)** | | | | | | | | | |
| ***Plasmodium vivax*** | **1 (7-190)** | | | | | | | | | |
| ***Plasmodium yoelii yoelii*** | **1 (7-190)** | | | | | | | | | |
| ***Plasmodium chabaudi chabaudi*** | **1 (7-190)** | | | | | | | | | |
| ***Plasmodium berghei*** | **1 (7-190)** | | | | | | | | | |
| ***Toxoplasma gondii*** | **1 (7-191)** | | | | | | | | | |
| ***Cryptosporidium parvum*** | **1 (25-210)** | | | | | | | | | |
| ***Babesia bovis*** | **1 (8-192)** | | | | | | | | | |
| ***Theileria annulata*** | **1 (8-191)** | | | | | | | | | |
| ***Eimeria tenella*** | **1 (7-112)** | | | | | | | | | |
| ***Neospora caninum*** | **1 (7-191)** | | | | | | | | | |
| ***Theileria parva*** | **1 (8-191)** | | | | | | | | | |
| ***Phytophthora sojae*** | **1 (7-192)** | | | | | | | | | |
| **Sec23 (Human)**  **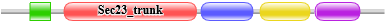**  **Zf Sec23 Sec24**  **Sec23 BS**  **Sec23 helical**  **Gelsolin** | | | | | | | | | | |
| **Organism** | **Zf Sec23 Sec24** | **Sec23**  **trunk** | | | | **Sec23 BS** | | **Sec23 helical** | **Gelsolin** | |
| ***Homo sapience*** | **1 (58-98)** | **1 (126-392)** | | | | **1 (403-506)** | | **1 (520-619)** | **1 (631-720)** | |
| ***Arabidopsis thaliana*** | **1 (57-95)** | **1 (124-392)** | | | | **1 (402-508)** | | **1 (522-621)** | **1 (633-722)** | |
| ***Saccharomyces cerevisiae*** | **1 (53-93)** | **1 (119-388)** | | | | **1 (399-512)** | | **1 (524-623)** | **1 (635-724)** | |
| ***Plasmodium falciparum*** | **1 (55-93)** | **1 (122-384)** | | | | **1 (394-497)** | | **1 (509-608)** | **1 (620-710)** | |
| ***Plasmodium knowlesi*** | **1 (55-93)** | **1 (122-383)** | | | | **1 (394-497)** | | **1 (509-608)** | **1 (620-710)** | |
| ***Plasmodium vivax*** | **1 (55-93)** | **1 (122-383)** | | | | **1 (394-497)** | | **1 (509-608)** | **1 (620-710)** | |
| ***Plasmodium yoelii yoelii*** | **1 (55-93)** | **1 (122-383)** | | | | **1 (394-497)** | | **1 (509-608)** | **1 (620-710)** | |
| ***Plasmodium chabaudi chabaudi*** | **1 (55-93)** | **1 (122-383)** | | | | **1 (394-497)** | | **1 (509-608)** | **1 (620-710)** | |
| ***Plasmodium berghei*** | **1 (55-93)** | **1 (122-383)** | | | | **1 (394-497)** | | **1 (509-608)** | **1 (620-710)** | |
| ***Toxoplasma gondii*** | **1 (53-91)** | **1 (131-407)** | | | | **1 (418-521)** | | **1 (533-632)** | **1 (646-735)** | |
| ***Cryptosporidium parvum*** | **1 (54-92)** | **1 (122-383)** | | | | **1 (394-497)** | | **1 (509-608)** | **1 (621-710)** | |
| ***Babesia bovis*** | **1 (58-96)** | **1 (127-376)** | | | **1 (387-512)** | | | **1 (524-623)** | **1 (635-725)** | |
| ***Theileria annulata*** | **1 (57-95)** | **1 (126-377)** | | | **1 (385-516)** | | | **1 (528-627)** | **1 (639-729)** | |
| ***Eimeria tenella*** | **1 (53-91)** | **1 (144-419)** | | | **1 (427-530)** | | | **1 (542-641)** | **1 (655-744)** | |
| ***Neospora caninum*** | **1 (53-91)** | **1 (133-410)** | | | **1 (421-524)** | | | **1 (536-635)** | **1 (649-738)** | |
| ***Theileria parva*** | **1 (57-95)** | **1 (126-377)** | | | **1 (385-516)** | | | **1 (528-627)** | **1 (639-729)** | |
| ***Phytophthora sojae*** | **1 (49-87)** | **1 (116-385)** | | | **1 (403-507)** | | | **1 (521-621)** | **1 (633-722)** | |
| **Sec24A (Human) 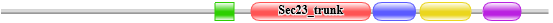**  **Zf Sec23 Sec24**  **Sec23 BS**  **Sec23 helical**  **Gelsolin** | | | | | | | | | | |
| **Organism** | **Zf Sec23 Sec24** | **Sec23**  **trunk** | | **Sec23 BS** | | | | **Sec23 helical** | **Gelsolin** | |
| ***Homo sapience*** | **1 (428-465)** | **1 (501-739)** | | **1 (744-828)** | | | | **1 (839-940)** | **1 (964-1039)** | |
| ***Arabidopsis thaliana* (Sec24)** | **1 (368-406)** | **1 (443-680)** | | **1 (685-769)** | | | | **1 (780-884)** | **1 (909-980)** | |
| ***Saccharomyces cerevisiae***  **(Sec24)** | **1 (228-265)** | **1 (301-548)** | | **1 (553-636)** | | | | **1 (647-750)** | **1 (780-856)** | |
| ***Plasmodium berghei*** | **1 (236-274)** | **1 (311-579)** | | **1 (585-669)** | | | | **1 (680-770)** | **1 (794-860)** | |
| ***Plasmodium chabaudi***  ***chabaudi*** | **1 (235-273)** | **1 (310-578)** | | **1 (584-668)** | | | | **1 (679-769)** | **1 (793-859)** | |
| ***Plasmodium falciparum*** | **1 (223-261)** | **1 (298-577)** | | **1 (582-666)** | | | | **1 (677-767)** | **1 (791-855)** | |
| ***Plasmodium knowlesi*** | **1 (224-262)** | **1 (299-570)** | | **1 (576-660)** | | | | **1 (671-759)** | **1 (788-849)** | |
| ***Plasmodium vivax*** | **1 (226-264)** | **1 (301-572)** | | **1 (578-662)** | | | | **1 (673-763)** | **1 (791-852)** | |
| ***Plasmodium yoelii*** | **1 (236-274)** | **1 (311-579)** | | **1 (585-669)** | | | | **1 (680-770)** | **1 (794-860)** | |
| ***Toxoplasma gondii*** | **1 (228-266)** | **1 (303-527)** | | **1 (601-699)** | | | | **1 (710-834)** | **1 (862-916)** | |
| ***Cryptosporidium parvum*** | **1 (196-234)** | **1 (271-516)** | | **1 (521-607)** | | | | **1 (618-710)** | **1 (732-800)** | |
| ***Eimeria tenella*** | **1 (126-157)** | **2 (194-409,**  **459-505)** | | **1 (510-603)** | | | | **1 (614-706)** |  | |
| ***Neospora caninum*** |  | **1 (16-125)** | | **1 (189-285)** | | | | **1 (296-420)** |  | |
| ***Theileria parva*** | **1 (232-270)** | **1 (306-550)** | | **1 (555-645)** | | | |  |  | |
| ***Theileria annulata*** | **1 (238-276)** | **1 (312-556)** | | **1 (561-651)** | | | |  |  | |
| ***Babesia bovis*** | **1 (179-217)** | **1 (251-484)** | | **1 (489-573)** | | | | **1 (584-676)** | **1 (698-763)** | |
| ***Phytophthora sojae*** | **1 (338-376)** | **1 (413-649)** | | **1 (654-738)** | | | | **1 (749-854)** | **1 (888-973)** | |
|  |  | **Zf Sec23 Sec24**  **Sec23 BS**  **Sec23 helical**  **Gelsolin** | |  | | | |  |  | |
| **Human Sec 24B 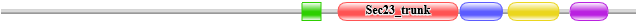** | | | | | | | | | | |
| **Organism** | **Zf Sec23 Sec24** | **Sec23**  **trunk** | | **Sec23 BS** | | | | **Sec23 helical** | **Gelsolin** | |
| ***Homo sapience*** | **1 (602-639)** | **1 (675-914)** | | **1 (919-1003)** | | | | **1 (1014-1115)** | **1 (1139-1214)** | |
| ***Plasmodium berghei*** |  | **1 (754-1019)** | | **1 (1025-1113)** | | | | **1 (1124-1222)** |  | |
| ***Plasmodium chabaudi***  ***chabaudi*** |  | **1 (754-1019)** | | **1 (1025-1113)** | | | | **1 (1124-1222)** |  | |
| ***Plasmodium falciparum*** |  | **1 (751-987)** | | **1 (993-1081)** | | | | **1 (1092-1190)** |  | |
| ***Plasmodium knowlesi*** |  | **1 (926-1169)** | | **1 (1176-1264)** | | | | **1 (1275-1373)** |  | |
| ***Plasmodium vivax*** |  | **1 (944-1192)** | | **1 (1198-1286)** | | | | **1 (1297-1395)** |  | |
| ***Plasmodium yoelii*** |  | **1 (745-1010)** | **1 (1016-1104)** | | | | | **1 (1115-1213)** |  | |
| ***Toxoplasma gondii*** | **1 (682-720)** | **1 (848-1148)** |  | | | | | **1 (1268-1366)** |  | |
| ***Cryptosporidium parvum*** | **1 (313-351)** | **1 (396-615)** | **1 (687-776)** | | | | | **1 (839-912)** |  | |
| ***Eimeria tenella*** | **1 (673-711)** | **1 (853-1019)** |  | | | | | **1 (1209-1309)** |  | |
| ***Neospora caninum*** | **1 (661-699)** | **1 (831-1126)** |  | | | | | **1 (1246-1344)** |  | |
| ***Theileria parva*** | **1 (292-329)** | **1 (446-669)** | **1 (674-762)** | | | | | **1 (773-856)** |  | |
| ***Theileria annulata*** | **1 (291-328)** | **1 (451-686)** | **1 (691-779)** | | | | | **1 (790-869)** |  | |
| ***Babesia bovis*** | **1 (245-281)** | **1 (425-660)** | **1 (665-754)** | | | | | **1 (766-853)** |  | |
| ***Phytophthora sojae*** | **1 (358-396)** | **1 (433-675)** | **1 (680-770)** | | | | | **1 (781-897)** |  | |

**Table S15:** The list of proteins identified to have interaction and co-expression for COPI and COPII.

| COPI | | COPII | |
| --- | --- | --- | --- |
| First shell of interaction | | First shell of interaction | |
| Interacting protein from String | New ID (Name of the protein) | Interacting protein from String | New ID (Name of the protein) |
| PFD0745c | PF3D7_0415400 (coatomer subunit zeta, putative) | PF13_0324 | PF3D7_1361100 (protein transport protein Sec24A) |
| PFF0330w | PF3D7_0606700 (coatomer alpha subunit, putative) | Sar1 | PF3D7_0416800 (small GTP-binding protein sar1) |
| PF11_0463 | PF3D7_1145100 (coatomer subunit gamma, putative) | PFL1480w | PF3D7_1230700 (protein transport protein SEC13) |
| PF14_0277 | PF3D7_1429800 (coatomer subunit beta, putative) | Pfsec23 | PF3D7_0822600 (protein transport protein SEC23) |
| PFI0290c | PF3D7_0905900 (coatomer subunit beta, putative) | Sec31p | PF3D7_0214100 (protein transport protein SEC31) |
| PF11_0359 | PF3D7_1134800 (coatomer subunit delta) | PFI0250c | PF3D7_0905100 (nucleoporin NUP100/NSP100, putative) |
| MAL8P1.121 | PF3D7_0808400 (coatomer subunit epsilon, putative) | PFD0872w | PF3D7_0418000 (conserved Plasmodium protein, unknown function) |
| PFF0655c | PF3D7_0613500 (AP-3 complex subunit beta, putative) | PfSec22 | PF3D7_0320100 (protein transport protein SEC22) |
| PFE1400c | PF3D7_0528100 (AP-1 complex subunit beta, putative) | PFD0250c | PF3D7_0405100 (protein transport protein Sec24B, putative) |
| MAL7P1.164 | PF3D7_0730200 (AP-4 complex subunit beta, putative) | PfSyn5 | PF3D7_1332000 (syntaxin, Qa-SNARE family) |
| PF08_0120 | PF3D7_0804900 (GTPase-activating protein, putative) | ERD2 | PF3D7_1353600 (ER lumen protein retaining receptor) |
| MAL13P1.163.1 | PF3D7_1330400 (ER lumen protein retaining receptor 1, putative) | Rab1a | PF3D7_0513800 (ras-related protein Rab-1A) |
|  |  | Rab1b | PF3D7_0512600 (ras-related protein Rab-1B) |
| Second shell of interaction | | Second shell of interaction | |
| PFB0805c | PF3D7_0217300 (AP-2 complex subunit sigma, putative) | PfVAMP8 | PF3D7_1303200 (SNARE protein, putative) |
| PFL2425w | PF3D7_1250500 (AP-3 complex subunit sigma, putative) | PFB0750w | PF3D7_0216400 (vacuolar protein sorting-associated protein 45, putative) |
| PF14_0529 | PF3D7_1455500 (AP-1 complex subunit gamma, putative) | PFE0445c | PF3D7_0509000 (alpha-soluble NSF attachment protein, putative) |
| PF13_0062 | PF3D7_1311400 (AP-1 complex subunit mu-1) | PFC0140c | PF3D7_0303000 (N-ethylmaleimide-sensitive fusion protein) |
| PFF0830w | PF3D7_0617100 (AP-2 complex subunit alpha, putative) | Syn16 | PF3D7_1243000 (syntaxin, Qa-SNARE family) |
| PF11_0202 | PF3D7_1119500 (AP-4 complex subunit mu, putative) | Syn17 | PF3D7_0210700 (syntaxin, Qa-SNARE family) |
| PFL2220w | PF3D7_1246300 (conserved Plasmodium protein, unknown function) | PFF0665c | PF3D7_0613700 (syntaxin-binding protein, putative) |
| PFD1090c | PF3D7_0423100 (AP-4 complex subunit sigma, putative) | PF10_0331 | PF3D7_1034000 (Sec1 family protein, putative) |
| PFI0200c | PF3D7_0904100 (AP-4 complex subunit epsilon, putative) | PfYkt6.1 | PF3D7_0910600 (SNARE protein) |
| PFL0930w | PF3D7_1219100 (clathrin heavy chain, putative) | PfYkt6.2 | PF3D7_1324700 (SNARE protein, putative) |
