# Supplementary figures and images for: A genome-wide analysis of coatomer protein (COP) subunits of apicomplexan parasites and their evolutionary relationships
Source: BMC Genomics. 2019 Jan 31;20:98. doi: 10.1186/s12864-019-5463-1 (PMC6357402; doi:10.1186/s12864-019-5463-1)

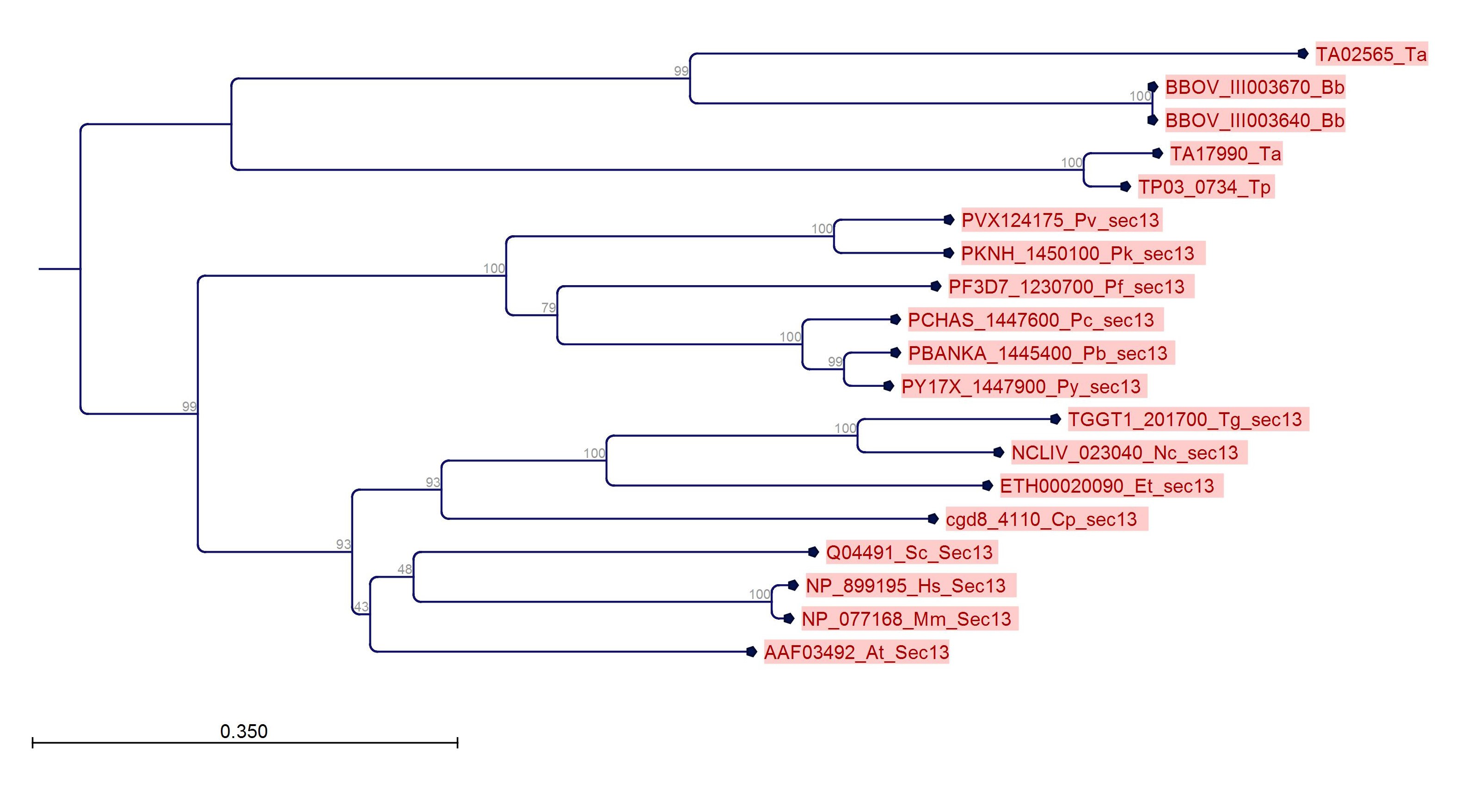

Supplement: Supplementary file 2 — Figure S1. The phylogenetic tree showing the relation between the T. annulata, T. parva and B. bovis putative sec13 proteins with other apicomplexan and higher order organisms. (TIF 1427 kb) [file 12864_2019_5463_MOESM2_ESM.tif]

**A.**

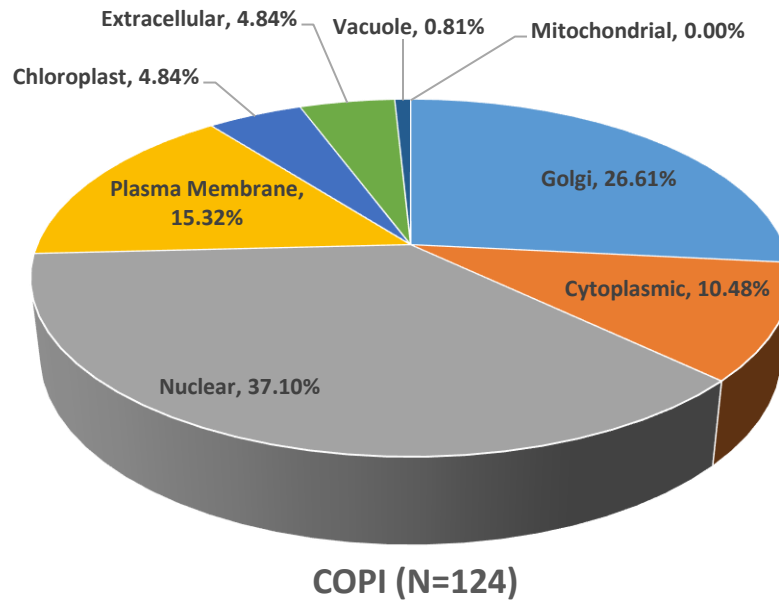

**B.**

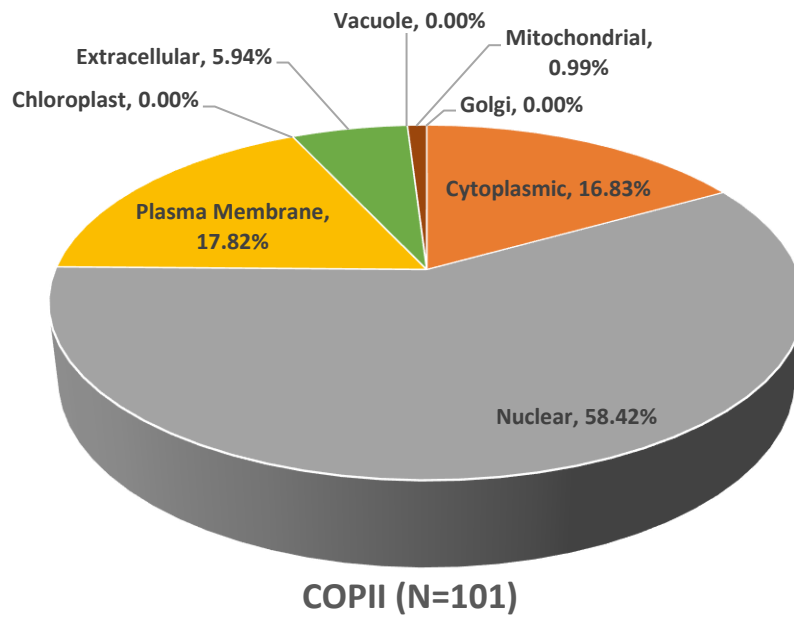

Supplement: Supplementary file 4 — Figure S6. Subcellular localization of the COPI (A) and COPII (B) proteins. (PDF 230 kb) [file 12864_2019_5463_MOESM4_ESM.pdf]

A.

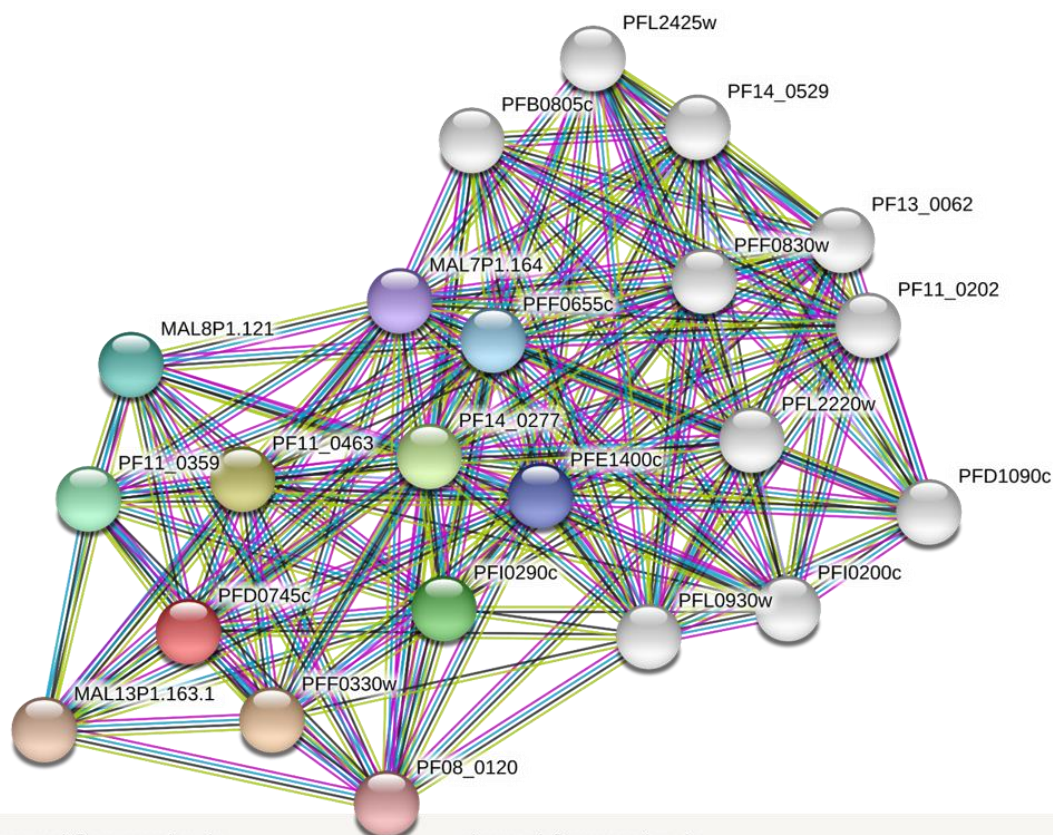

B.

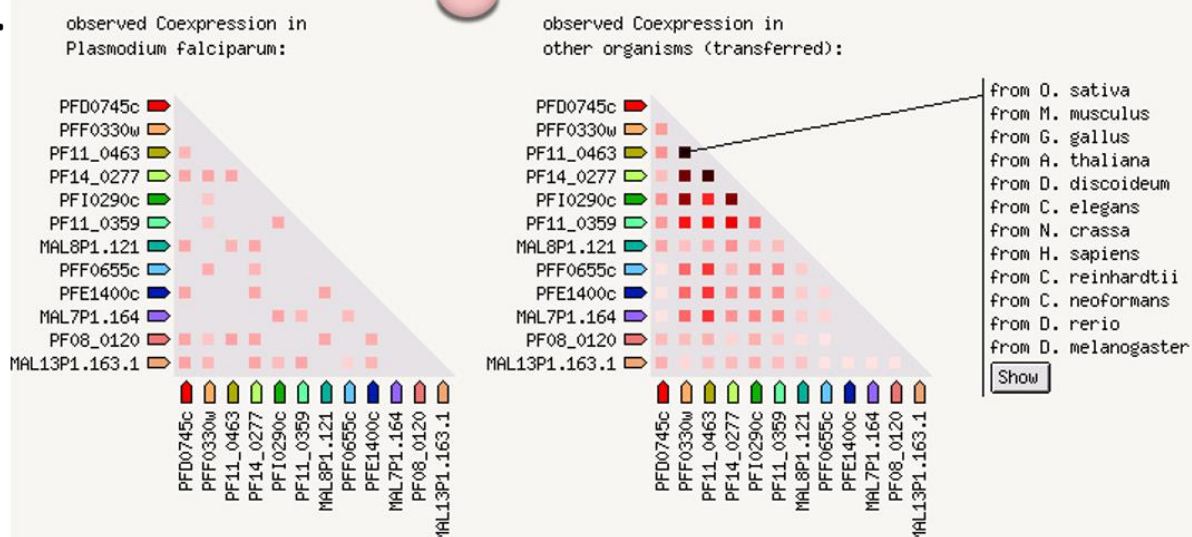

Supplement: Supplementary file 5 — Figure S2. Protein-protein interaction data of COPI retrieved from String. A. The first shell of interaction with COPI components are shown by colored nodes and the second shell of interaction are shown by white nodes. B. The proteins that are co-expressed in Plasmodium falciparum has been shown in first graph and the co-expression in other organisms are shown in second graph. The pink color edges are indicating experimentally proved interactions. (PDF 578 kb) [file 12864_2019_5463_MOESM5_ESM.pdf]

A.

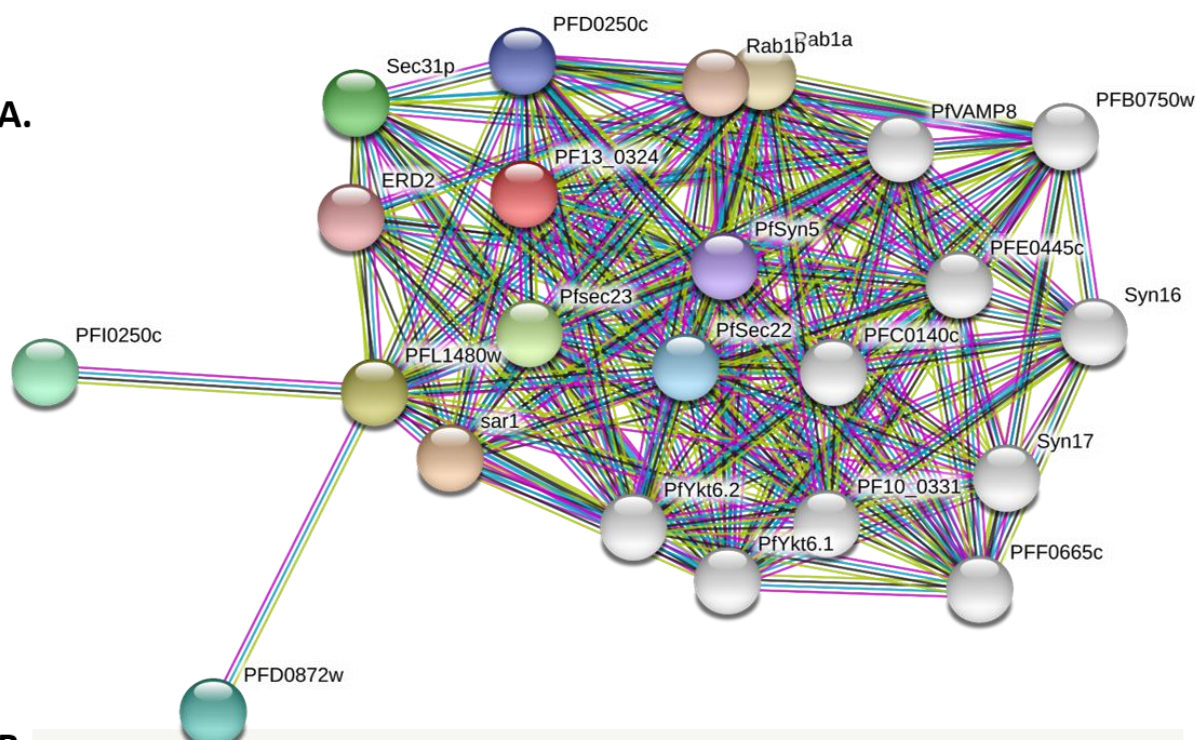

B.

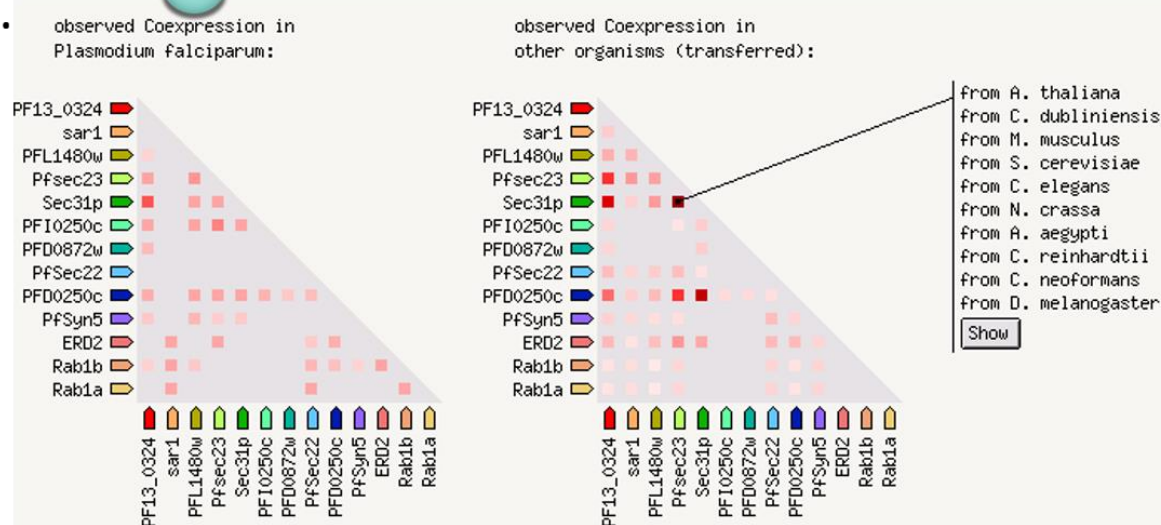

Supplement: Supplementary file 6 — Figure S3. Protein-protein interaction data of COPII retrieved from String. A. The first shell of interaction with COPII components are shown by colored nodes and the second shell of interaction are shown by white nodes. B. The proteins that are co-expressed in Plasmodium falciparum has been shown in first graph and the co-expression in other organisms are shown in second graph. The pink color edges are indicating experimentally proved interactions. (PDF 533 kb) [file 12864_2019_5463_MOESM6_ESM.pdf]

**A.**

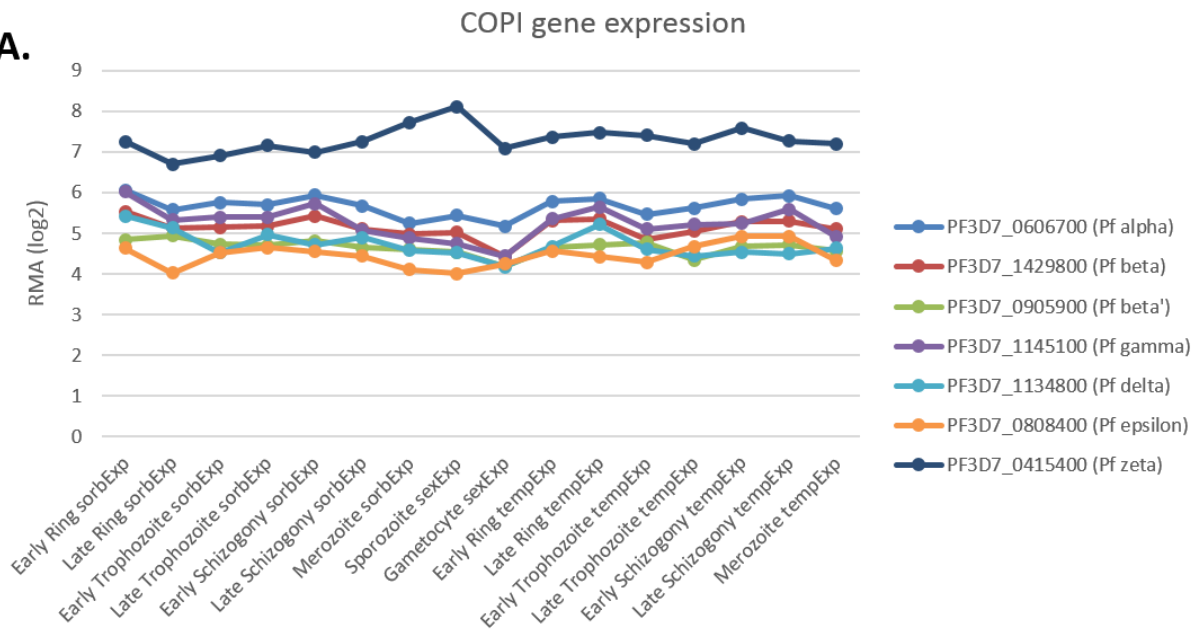

**B.**

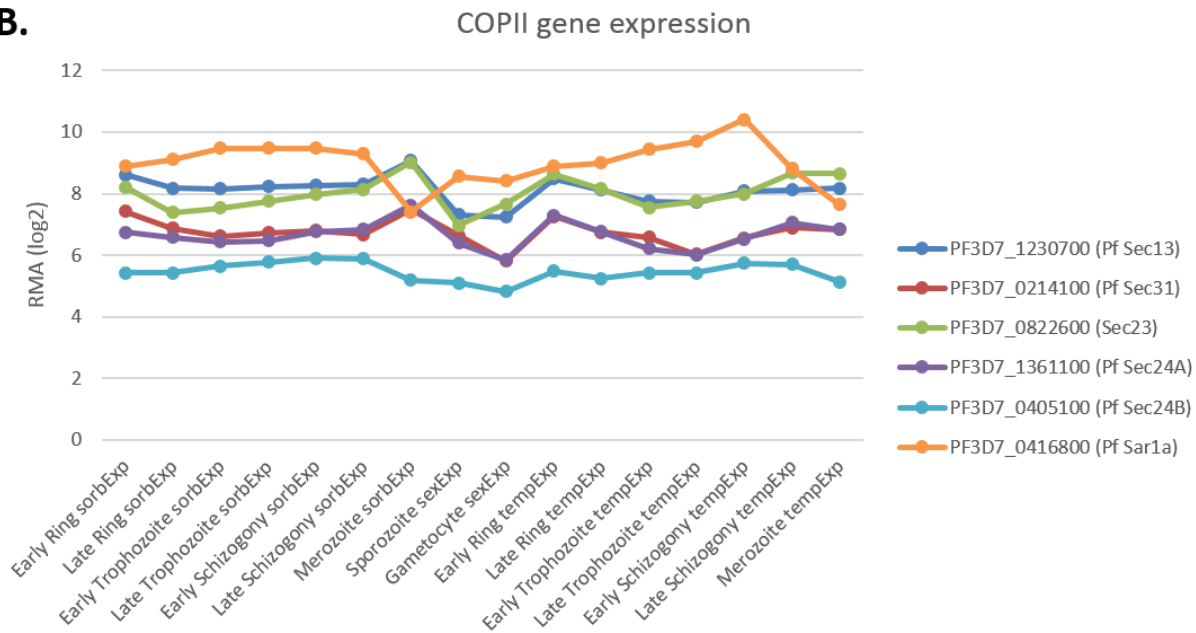

Supplement: Supplementary file 8 — Figure S4. The gene expression of COPI and COPII in different stages of Malaria parasite. The X-axis indicating different stages of malaria parasite and the Y-axis shows the normalized RMA value (log base 2). (PDF 243 kb) [file 12864_2019_5463_MOESM8_ESM.pdf]

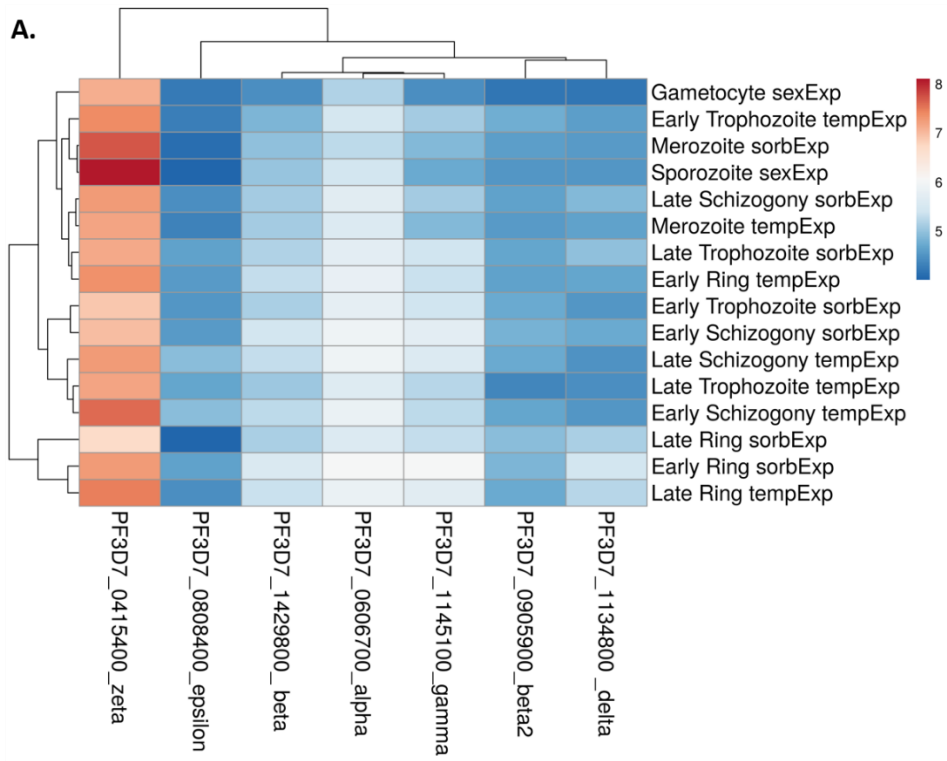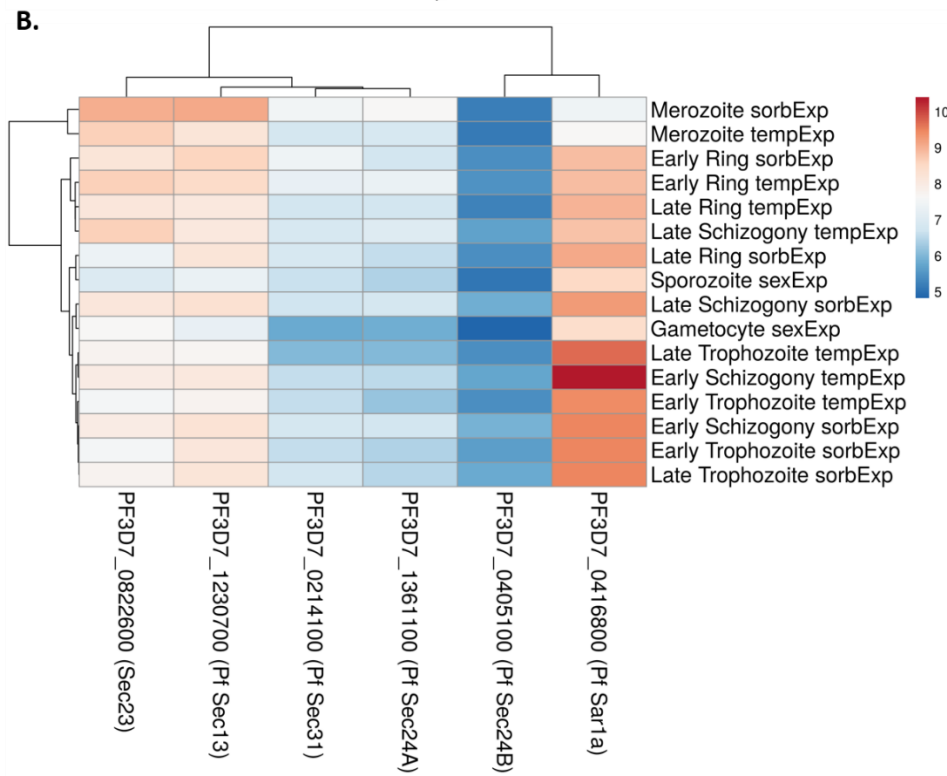

Supplement: Supplementary file 9 — Figure S5. The heat map showing gene expression profiles of COPI and COPII in different stages of Malaria parasite. (PDF 427 kb) [file 12864_2019_5463_MOESM9_ESM.pdf]
